# The innate immune mediator group IIA secreted phospholipase A_2_ modulates lipid droplet formation in prostate cancer cells

**DOI:** 10.3389/fcell.2026.1774027

**Published:** 2026-05-18

**Authors:** Monavvar Andarva, Maria George Elias, Mila Sajinovic, Timothy J. Mann, Jasmine M. Zhou, Albert S. Mellick, Tara Laurine Roberts, Paul de Souza, Kieran F. Scott

**Affiliations:** 1 School of Medicine, Faculty of Health, Western Sydney University, Campbelltown, NSW, Australia; 2 Medical Oncology Unit, Ingham Institute of Applied Medical Research, Liverpool, NSW, Australia; 3 Nepean Hospital Clinical School, Faculty of Medicine and Health, University of Sydney, Kingswood, NSW, Australia; 4 School of Biomedical Sciences, UNSW, Kensington, NSW, Australia; 5 Graduate School of Biomedical Engineering, UNSW, Kensington, NSW, Australia

**Keywords:** BODIPY, DGAT, FASN, immunofluorescence, kesonotide, PLIN2, PLIN3, vimentin

## Abstract

**Introduction:**

We and others have shown that elevated human Group IIA secreted phospholipase A_2_ (hGIIA) has both catalysis-dependent and -independent functions that are associated with both acute and chronic inflammatory disorders including cancer. Our novel inhibitors that selectively block hGIIA’s catalysis-independent function slow tumor growth in animal models via interaction with vimentin. The mechanism of this interaction and its consequences for cancer aggressiveness remains unexplored.

**Methods:**

We have used established patient-derived androgen-independent prostate cancer (PCa) cell lines, DU145, PC-3 and vimentin-knockout DU145 (DU145^vim−^) cells treated with hGIIA protein in the presence and absence of our cyclic peptide inhibitor Kesonotide (also known as c2). As lipid metabolism is known to be regulated by vimentin, we examined whether this aspect of vimentin biology was influenced by interaction with hGIIA, focusing on lipid droplet (LD) formation, LD-associated perilipin proteins (PLIN2 and PLIN3) and lipid biosynthetic enzymes (DGAT and FASN). To do this we used the combination of single cell quantitative immunofluorescence and Western blot analysis.

**Results:**

We found that addition of exogenous hGIIA increased LD metabolism as evidenced by LD immunofluorescence signal and this increase is dependent on vimentin in PCa. The response is dependent on hGIIA levels with high concentrations of hGIIA associated with PLIN2 loss, PLIN3 suppression, and DGAT1 induction without FASN upregulation. Kesonotide alone was largely inert. However, at high concentration of hGIIA, Kesonotide attenuated lipid metabolism by neutralizing perilipin remodelling and attenuating DGAT1 induction in knockout cells.

**Discussion:**

Taken together, these findings identify a context-dependent hGIIA LD-remodelling program that is shaped by vimentin, implicating for the first time the hGIIA–vimentin axis in inflammatory cue–driven lipid metabolic rewiring in aggressive PCa.

## 1 Introduction

Human group IIA secreted phospholipase A_2_ (hGIIA, human PLA2G2A) is a well-known driver of innate immunity and inflammation (Scott et al., 2021). Work from our laboratory and others has also now drawn a link between the aggressiveness of cancer cells and the non-lipase activity of hGIIA (Mann et al., 2026). The mechanism of this novel pathway and a link with cancer malignancy is yet to be fully explored.

The last several decades have led to a dramatic improvement in survival of men diagnosed with prostate cancer (PCa) in developed countries from ∼65% 5-year survival in the 1980s to over 90% 10 years survival in 2025 (AIHW Web Report, 2025; Kratzer et al., 2025). Despite this improvement, PCa remains the second largest cause of cancer deaths in men behind lung cancer approaching 400,000 deaths in 2022 worldwide (AIHW Web Report, 2025; World Cancer Research Fund, 2025). The absolute number of deaths continues to rise due largely to global population growth and population ageing.

It is now clear that PCa is a highly heterogeneous disease at a molecular level and while many PCa tumors grow slowly, a subset of patients (15%–20%) have tumors that are aggressive (Rebello et al., 2021). These aggressive tumors do not respond well to standard androgen deprivation therapy. While current therapies control metastatic disease, once metastasized, particularly to bone, the disease is essentially incurable. PCa cells are also highly adaptive to external environmental pressure, resulting in the development of resistance to both endogenous immune surveillance and drug treatments. Such evolutionary pressure results in castrate resistant PCa, responsible for most PCa deaths (Gundem et al., 2015). As a result there remains a need to identify and validate novel targets and approaches to treating advanced PCa phenotypes (Bernal et al., 2024).

hGIIA has long been one such target due to its: (i) aberrant expression in prostate tumors (Sved et al., 2004) and well-documented roles in the innate immune response as an antibacterial protein in the gut (Nevalainen et al., 2008; Doré, et al., 2022) and as an amplifier of cytokine-mediated inflammatory responses both *in vitro* (Bidgood et al., 2000) and in animal models of chronic inflammatory diseases such as rheumatoid arthritis (Boilard et al., 2010). Early studies established that elevated hGIIA protein expression in prostate tumors relative to matched normal tissue was associated with poorer outcomes (Sved et al., 2004 and references therein), while recent transcriptomics data suggests that in large patient cohorts, *PLA2G2A* expression is associated with better outcomes (Recuero et al., 2024), indicating that hGIIA effects are highly context-dependent with epithelial cells showing a negative correlation while stromal and immune cells show a positive correlation in aggressive disease contexts (Chen et al., 2022; Chen et al., 2021; Li et al., 2024).

Interestingly, we and others have shown that hGIIA is a bifunctional protein which, in addition to its phospholipase catalytic activity interacts with both intracellular and extracellular proteins, consequently modulating intracellular signalling pathways (Bryant et al., 2011; Scott et al., 2021). This second function does not require hGIIA catalytic activity (Lee et al., 2013). In PCa cells, we have demonstrated that exogenous hGIIA, through either autocrine secretion or by paracrine release from innate immune cells present in the tumor microenvironment, binds directly to tumor cell epidermal growth factor receptor (EGFR) resulting in activation of the ERK mitogen activated kinase pathway, subsequent phosphorylation of cytosolic phospholipase A_2_-α and elevation of the growth-promoting eicosanoid prostaglandin E_2_ (PGE_2_). In addition, following internalization via caveolae, hGIIA-containing vesicles are trafficked intracellularly by binding to vimentin resulting in lower vesicle tracking speed and particle size relative to vimentin-unbound vesicles (Mann et al., 2026).

Despite extensive clinical trials of inhibitors that potently target hGIIA catalytic activity no hGIIA inhibitors have been approved for clinical use (Scott et al., 2021). The most promising inhibitor reached Phase III trial in cardiovascular disease but was prematurely halted due to safety concerns and lack of efficacy (Nicholls et al., 2014). We have designed a cyclic pentapeptide, cyclo-(2-Nal)-Leu-Ser-(2-Nal)-Arg, (c2, also known as Kesonotide) derived from the primary sequence of hGIIA that, unlike prior designed hGIIA inhibitors, selectively inhibits the catalysis-independent function of hGIIA (Lee et al., 2013). We have shown that this compound, on oral delivery, suppresses tumor growth in three independent xenograft models of PCa, in some cases resulting in complete tumor regression (Mann et al., 2026). In PCa cells, c2 inhibits the hGIIA/EGFR interaction, reducing EGFR, ERK and cPLA-α phosphorylation together with cytokine-dependent PGE_2_ production, while slowing hGIIA trafficking within the cell, disrupting the hGIIA-vimentin interaction, resulting in aggresome formation and vimentin-dependent apoptosis (Mann et al., 2026). These data prompted us to examine whether hGIIA-vimentin and/or c2 could regulate lipid metabolic reprogramming in PCa cells.

Lipid metabolic reprogramming is a hallmark of cancer (Beloribi-Djefaflia et al., 2016). The demand for increased energy to drive increased proliferation is met, in part, by upregulation of exogenous (dietary) lipid and cholesterol uptake pathways together with aberrant induction of endogenous *de novo* lipid and cholesterol synthesis pathways driven by increased glucose and glutamine uptake and metabolism. These triglyceride lipids and cholesterol esters are stored in lipid droplets (LDs). High LDs and stored cholesterol ester content in tumors are thus indicators of tumor aggressiveness.

Studies in non-cancer cell types, particularly differentiating adipocyte cells, have shown that vimentin regulates LD biogenesis, fatty acid uptake, fatty acid oxidation, lipid trafficking and mitochondrial fatty acid oxidation (Leiber and Evans, 1996; Heid et al., 2014; Shen et al., 2010; Hirata et al., 2011). Notably, an *in vivo* role for vimentin in regulating body fat in mammals has been demonstrated in vimentin knockout mice fed both normal or high-fat fed diets (Wilhelmsson et al., 2019; Kim et al., 2021).

To the best of our knowledge there is no direct evidence for a role for vimentin in PCa lipid metabolic reprogramming nor for a role of hGIIA in regulating reprogramming. In light of the knowledge gap in this area, this work evaluates for the first time the effect of exogenous hGIIA on lipid metabolic reprogramming in androgen-resistant PCa cell lines DU145 and PC-3, the effect of vimentin deletion on metabolic reprogramming and the effect of the hGIIA/vimentin protein-protein interaction inhibitor Kesonotide on reprogramming in these cell lines.

## 2 Materials and methods

All chemicals used in this study were analytical grade. Androgen-independent PCa cell lines PC-3 and DU145 were obtained from ATCC (Manassas VA). Cell line DU145^vim−^ was constructed by CRISPR/Cas9 deletion as described (Mann et al., 2026). Cell line identity was confirmed by short tandem repeat analysis (data not shown) and used at passage numbers less than 20.

hGIIA was purified by affinity chromatography using an AKTA Smart FPLC (GE Healthcare, Rydalmere NSW, Australia) from conditioned media obtained from 25L culture of hGIIA-expressing Chinese hamster ovary cell line 2B1 (Bidgood et al., 2000) grown on alginate beads. Kesonotide (cyclo-(2-Nal)-Leu-Ser (2-Nal)-Arg), was a kind gift from Filamon Ltd (https://www.filamon.com).

Cells were maintained at 37 °C, 5% CO_2_, humidified atmosphere in RPMI-1640 (Life Technologies, Mulgrave, Victoria) containing 10% FBS (Scientifix, Clayton, VIC, Australia) and 1% penicillin-streptomycin (Life Technologies). Cell lines were confirmed as mycoplasma negative following monthly testing using Mycoalert Mycoplasma kit (Lonza, Norwest, NSW, Australia).

To determine the effect of exogenous hGIIA on LD formation, cells were seeded (7,000 cells/well) into glass-bottom 96-well plates (Phenoplate, Revvity, Scoresby VIC. Australia). Once cultures reached ∼80% confluency, cells were treated, each in triplicate, with vehicle or hGIIA at 10 nM or 100 nM for 72 h.

LDs were visualized, following treatment, by staining with the lipid-selective neutral dye BODIPY TM 558/568 C12 (Thermo Fisher Scientific, Scoresby, VIC., Australia) as follows. Cells were rinsed with phosphate-buffered saline (PBS, Thermo Fisher Scientific) and fixed (4% paraformaldehyde in PBS, 20 min at room temperature). Following two PBS washes, LDs were stained for 10 min at 37 °C with BODIPY (2 μM in PBS). Cells were washed gently 2–3 times with PBS and mounted with PBS. Images were acquired on a laser-scanning confocal microscope (LSM800, Carl Zeiss, Macquarie Park, NSW, Australia) using a ×20 objective. BODIPY fluorescence was captured with 559/568 excitation/emission settings and identical laser power, detector gain/offset, pinhole, pixel size, dwell time, and z-position across conditions within each biological replicate.

LDs were quantified using artificial intelligence-based Aivia software (Version 14.0.0.41095, Leica, Mt Waverley, VIC., Australia) with the following concise, image-by-image segmentation workflow. In the segmentation phase, for each field, the AI segmentation pipeline in Aivia was applied to the BODIPY channel to delineate cell masks and LD signal. Pipeline parameters were fixed within each biological replicate and reused across treatment conditions to ensure consistency. Segmentation results were visually reviewed image by image. Objects corresponding to half/edge cells, merged or over-segmented cells, and obvious mis-segmentations (e.g., out-of-focus cells or debris) were excluded. When needed, minor edits were applied to correct boundaries. For the remaining, high-confidence objects, mean BODIPY intensity per segmented cell was calculated and exported. Cell-level values were averaged to a per-field value; fields were then averaged to a per-well value; wells (n = 3 technical replicates) were averaged to a per-experiment value for each treatment. Group means and variability were then calculated across biological replicates (n = 3). This image-by-image AI segmentation with explicit exclusion of partial and incorrectly segmented cells yielded precise segmentation and robust mean BODIPY intensity quantification while minimizing bias from edge effects and segmentation artifacts.

Western immunoblotting was performed as follows. Cells were seeded in 6-well plates at ∼1.0 × 10^6^ cells/well and allowed to adhere overnight. Cells were treated for 72 h with human group IIA secreted phospholipase A_2_ (hGIIA; 10 or 100 nM) or with Kesonotide (100 μM, 50 μM, 10 μM, 1 μM, 10 nM). For hGIIA, untreated cells in medium alone served as control. For Kesonotide, controls were medium containing 0.5% (v/v) dimethylsulfoxide (DMSO) for the 100 μM and 50 μM doses and 0.1% (v/v) DMSO for the 10 μM, 1 μM, and 10 nM doses. Medium was refreshed every 24 h for all conditions. After 72 h, wells were washed twice with ice-cold phosphate-buffered saline (PBS) and lysed on ice in RIPA buffer (150 mM NaCl, 0.1% Triton X-100, 0.5% sodium deoxycholate, 0.1% sodium dodecyl sulfate (SDS), 50 mM Tris-HCl pH 8.0), supplemented with protease inhibitor cocktail tablets according to the manufacturer’s instructions (Roche, North Ryde, NSW, Australia). Lysates were harvested by scraping, incubated for 15 min on ice, and clarified (14,000 × g, 15 min, 4 °C). Supernatants were collected, protein concentration determined by BCA assay (Bio-Rad, South Granville, NSW, Australia) and samples stored at −80 °C until use.

Cell lysates were mixed 1:3 with 4 × sample buffer (Laemmli buffer, Bio-Rad), Bolt™ sample reducing agent (10x, ThermoFisher) and denatured for 10 min at 70 °C. Samples were briefly centrifuged prior to loading. Molecular weight markers (Spectra Multicolor Broad Range Protein Ladder (10–260 kDa, ThermoFisher) were loaded in at least one outer lane of every gel. Samples (20–30 µg total protein) were subjected to SDS polyacrylamide gel electrophoresis on Bolt™ 4%–12% Bis-Tris Plus gels with 1 × MES SDS running buffer (ThermoFisher) at 120 V. To minimize lane-position bias, lane order was randomized within genotype and condition; a vehicle/untreated control lane was included on every gel for normalization. After electrophoresis, gels were immediately prepared for PVDF transfer.

Prior to transfer, membranes (PVDF, ThermoFisher))were blocked in 5% (w/v) BSA in Tris buffered saline containing 0.5% Triton X-100 pH 7.0 (TBST) for 1 h at room temperature. Membranes were incubated with primary antibody for 24 h at 4 °C. All primary and secondary antibodies were diluted in 5% bovine serum albumin (BSA)/TBST. Primary antibodies used in this study were rabbit monoclonal anti-human PLIN2 (Cell Signaling Technology, Danvers, MA, clone E6G6M, catalog #95109, 1:1000 dilution), mouse anti humanTIP47/PLIN3 monoclonal, (Proteintech, Rosemount IL, #66523-1-Ig, 1:5000), Rabbit anti-human FASN monoclonal (Cell Signaling Technology, clone C20G5, #3180, 1:1000), rabbit antihuman DGAT1 monoclonal (Proteintech, 82945-1-RR, 1:2000). Rabbit anti-human GAPDH monoclonal (Cell Signaling Technology, clone 14C10, #2118, 1:1000) was used as a loading control for membranes probed with mouse primary antibodies. Mouse anti-human GAPDH monoclonal (Abcam, Melbourne, VIC, Australia, clone 6C5, #ab8245, 1:100,000) was used as a loading control for membranes probed with rabbit primary antibodies. Following primary antibody incubation, membranes were washed in TBST (3 × 10 min washes) and incubated with HRP-linked secondary antibodies for1 h at room temperature). Secondary antibodies used were goat anti-rabbit IgG-HRP (Cell Signaling Technology, #7074, 1:1000) and horse anti-mouse IgG-HRP, (Cell Signaling Technology, #7076, 1:1000) Membranes were imaged following incubation with chemiluminescence reagent (Plus-ECL, PerkinElmer, Waltham, MA) for 7 min on a LI-COR Odyssey® FC imaging system (LI-COR Biosciences, Lincoln, NE) in chemiluminescence mode within the linear dynamic range. Bands were quantified using LI-COR Image Studio, v5.2. For each lane, target intensities were normalized to the matched GAPDH loading control used on that membrane. For cross-blot comparison, normalized values were scaled to the matched control within each condition. For each lane, target intensities were normalized to the species-matched GAPDH loading control used on that membrane.

The expression of PLIN2 in single cells was visualized by immunofluorescence (IF) using a rabbit anti-human monoclonal PLIN2 primary antibody (Proteintech, #80362-2-RR, 1:250) detected with a CoraLite® Plus 647-conjugated goat anti-rabbit IgG polyclonal antibody (Proteintech, Cat no., RGAR005, 1:200). PLIN3 expression was visualized by IF using a rabbit anti-human polyclonal antibody directly conjugated with CoraLite® 647 (Proteintech, Cat no: CL647-10694, 1:200). Cells were fixed and imaged as described above for BODIPY staining. Images were analysed using Aivia software as described above for LDs.

Image Acquisition, Quantification, Data Processing and Statistical Analysis. Imaging was performed on three independent biological replicates performed on separate occasions. In each biological replicate, each treatment was assessed in three wells, with one field of view acquired per well using identical imaging settings within the experiment. Mean BODIPY intensity was extracted at the single-cell level after AI-assisted segmentation and quality filtering using Aivia software (Version 14.0.0.41095). Images were segmented with per-image quality control by exclusion of half/edge cells, merged, or mis-segmented objects within a biological replicate. Over 200 cells that passed the quality control were retained for single cell analysis. Mean intensity per segmented cell was exported to GraphPad Prism and analyzed at the single-cell level for each cell line. Violin plots show the distribution of single-cell values of one representative biological replicate to show the distribution of intensities and were not used as the statistical unit. The three well means were averaged to obtain one value per treatment per biological replicate. Statistical analyses were performed on these biological replicate values (N = 3), whereas wells were treated as technical replicates. Analysis was performed using the Mann Whitney U non-parametric pairwise test for data sets that were not normally distributed as determined by the Shapiro-Wilk test. Raw data sets or log-transformed data sets that were normally distributed were analysed by two-tailed unpaired Student’s *t*-tests.

All comparisons for Western blot analyses were assessed using two-tailed unpaired Student’s t-tests. All graphs and statistical analysis was performed in GraphPad Prism (v10.2.2, GraphPad Software, La Jolla, CA).

## 3 Results

**FIGURE 1:**
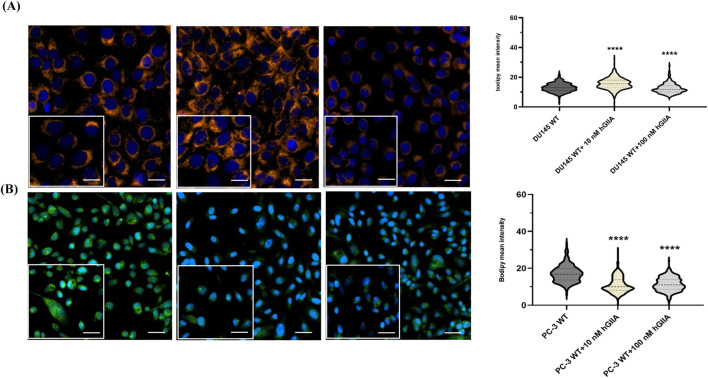
hGIIA differentially modulates lipid droplet content in prostate cancer cells. **(A)** Representative confocal images of DU145 cells treated for 72 h with vehicle, 10 nM or 100 nM hGIIA and stained with BODIPY 558/568 C12 to label LDs (orange) and DAPI to label nuclei (blue). Right: violin plots showing the distribution of single-cell BODIPY mean intensity quantified using AI-assisted segmentation in Aivia from three independent experiments, each with three technical replicates as described in Materials and Methods. **(B)** Representative images and corresponding violin plots for PC-3 cells treated as in **(A)**, with BODIPY shown in (pseudocolor green) and nuclei in blue. Data are representative images and violin plots of three independent experiments. ****p < 0.0001 (Mann Whitney U test) relative to baseline control. Scale bars 20 μm. Inset 10 μm^2^.

We first examined how hGIIA affects neutral lipid storage compartments in PCa cells using BODIPY558/568 C12 staining and AI-assisted image segmentation. In DU145 cells, untreated controls displayed numerous perinuclear LDs together with diffuse cytoplasmic staining (Figure 1A, left). Exposure to 10 nM hGIIA visibly increased the intensity of LD-associated BODIPY signal while 100 nM hGIIA led to a reduction in the signal relative to untreated control (Figure 1A, left). Quantification of single-cell BODIPY mean intensity confirmed a significant increase at 10 nM hGIIA and a significant decrease at 100 nM compared with untreated DU145 cells (Figure 1A, right). In PC-3 cells, untreated controls showed intense LD staining on a background of cytoplasmic staining. hGIIA treatment produced a strong suppressive response, with both 10 nM and 100 nM hGIIA lowering BODIPY fluorescence intensity relative to untreated controls and the strongest effect being observed at 10 nM (Figure 1B). Violin plot analysis of per-cell intensities showed significantly lower BODIPY mean intensity at both doses relative to untreated PC-3 cells (Figure 1B, right). Together, these data indicate that hGIIA alters neutral lipid accumulation in a dose- and cell line–dependent manner, enhancing LD-associated lipid content in DU145 at low dose while reducing in PC-3 cells.

**FIGURE 2:**
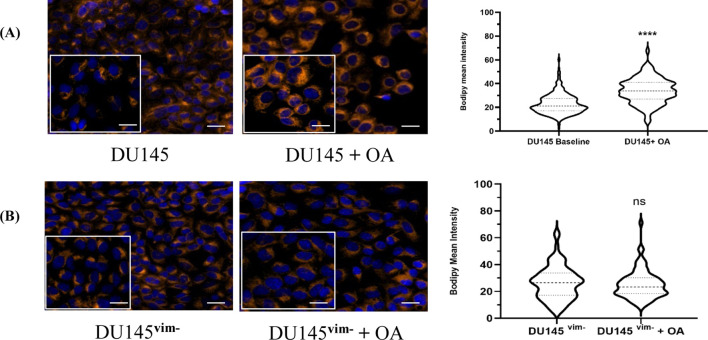
Oleic acid–induced lipid droplets in DU145 vs. DU145^vim−^ cells. BODIPY staining intensity of **(A)** WT DU145 cells or **(B)** DU145^vim−^ cells in the presence or absence of OA (50 μM) was measured and analysed as described in Material and Methods. Data are representative images and violin plots of three independent experiments. ****p < 0.0001 (Mann Whitney U test) relative to baseline control. Scale bars 20 μm. Inset 10 μm.

To dissect the role of vimentin in LD biogenesis, we next used a CRISPR/Cas9-engineered vimentin-knockout DU145 cell line (DU145^vim−^), previously generated and validated in our laboratory (Mann et al., 2026). We stimulated LD formation with oleic acid (OA) and quantified BODIPY staining in parental and vimentin-deficient cells. In parental DU145 cells as expected, OA treatment led to a clear increase in the intensity of LD-associated BODIPY signal compared with untreated controls (Figure 2A, left), and single-cell BODIPY mean intensity was significantly elevated in OA-treated cells (Figure 2A, right). In contrast, OA failed to enhance LD staining in DU145^vim−^ cells BODIPY signal and mean intensity distributions were similar between control and OA-treated conditions, with no significant difference detected (Figure 2B). These data indicate that vimentin is required for efficient OA-induced LD accumulation in DU145 cells.

**FIGURE 3:**
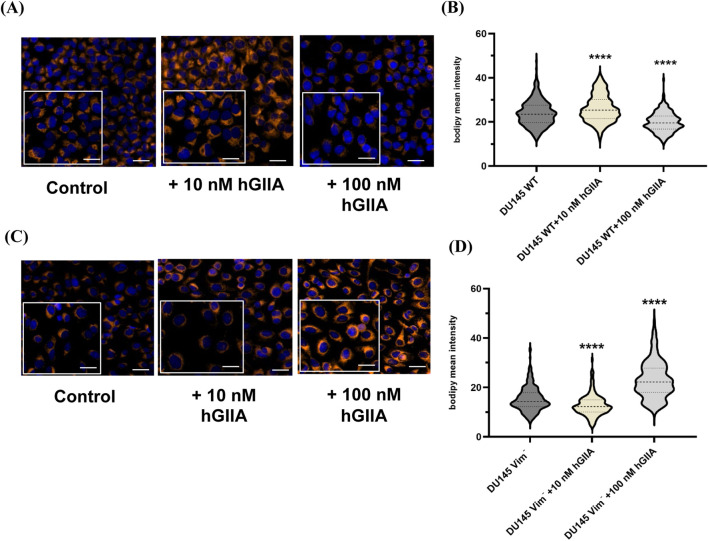
Vimentin tunes cellular sensitivity to hGIIA-induced lipid-droplet biogenesis. WT DU145 **(A,B)** and vimentin-knockout DU145^vim−^ cells **(C,D)** were treated for 72 h with vehicle, 10 nM or 100 nM hGIIA, then stained with BODIPY (orange) and DAPI (blue). **(A,C)** Representative confocal images; **(B,D)** violin plots of BODIPY mean intensity from three independent experiments. ****p < 0.0001 (Mann Whitney U test) versus baseline control captured and analysed as described in Materials and Methods. Scale bars 20 μm. Inset 10 μm.

Having established that vimentin is required for efficient oleic acid–driven LD biogenesis (Figure 2), we next asked whether vimentin also modulates LD remodelling in response to hGIIA. Parental DU145 and vimentin-knockout DU145^vim−^ cells were treated with 10 or 100 nM hGIIA and LD content was quantified by BODIPY staining. To directly compare genotype-specific dose–responses, we analysed images from a second biological replicate of parental DU145 cells (Figure 3A). In this cell line, hGIIA produced the strongest LD response at 10 nM, with a clear increase in BODIPY mean intensity compared with baseline, whereas 100 nM hGIIA caused a modest but statistically significant decrease relative to baseline (Figure 3B), consistent with Figure 1A.

In vimentin-knockout DU145^vim−^ cells, the pattern was inverted. 10 nM hGIIA reduced BODIPY fluorescence, while 100 nM hGIIA caused a marked accumulation of LDs (Figure 3C). Quantitative analysis showed a decrease in BODIPY mean intensity at 10 nM and a pronounced increase at 100 nM hGIIA in DU145^vim−^ cells (Figure 3D). Thus, vimentin is required for the LD-promoting effect of low-dose hGIIA and appears to restrain LD expansion at higher hGIIA concentrations; in its absence, the hGIIA dose–response is flipped, with suppression at low dose and exaggerated LD accumulation only at high dose.

**FIGURE 4:**
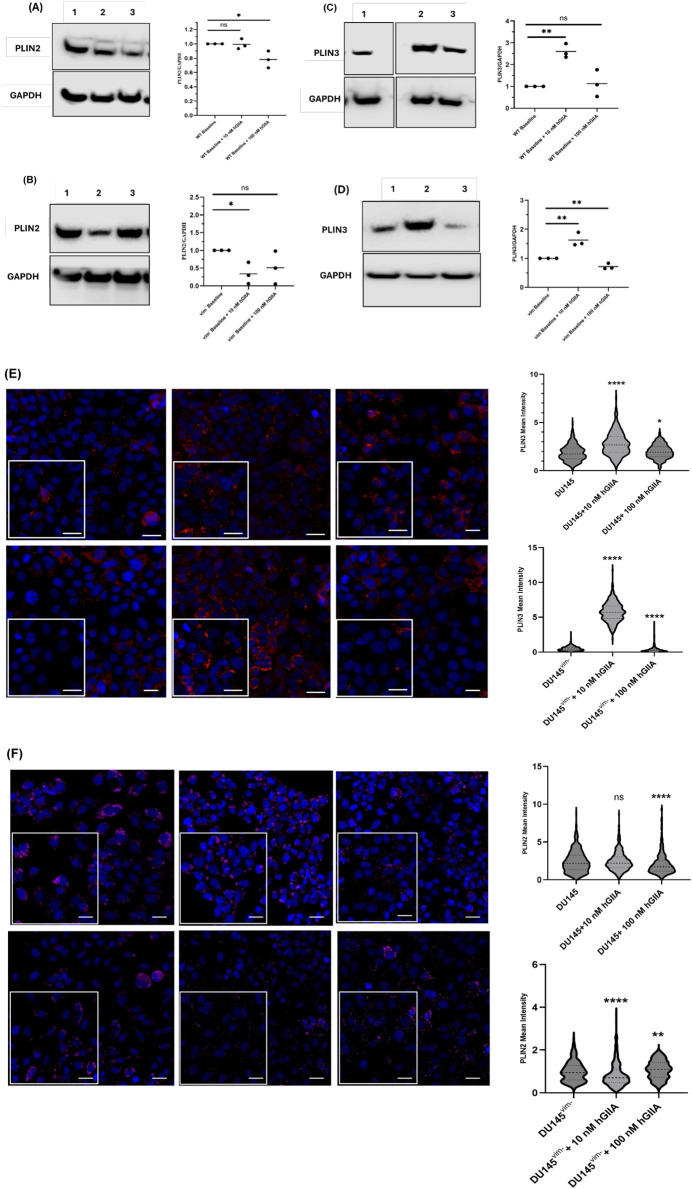
hGIIA differentially regulates PLIN2 and PLIN3 in DU145 WT and vimentin-knockout cells. **(A,B)** Representative western blots and quantification of PLIN2 normalized to GAPDH in DU145 WT **(A)** and DU145^vim−^ **(B)** cells treated for 72 h with vehicle (baseline), 10 nM or 100 nM hGIIA. **(C,D)** Representative blots and quantification of PLIN3 normalized to GAPDH in DU145 WT **(C)** and DU145^vim−^ **(D)** cells under the same conditions. Data points represent independent biological replicates (n = 3); horizontal lines indicate the mean. Statistical significance was assessed by unpaired Student’s t-test; ns, not significant; *p < 0.05; **p < 0.01. Uncropped Western blots, with their corresponding molecular markers, are shown in Supplementary Figure S1. **(E,F)** Confocal immunofluorescence of PLIN3 **(E)** and PLIN2 **(F)** in DU145 WT (top panels and upper violin plots) and DU145^vim−^ cells (bottom panels and lower violin plots) treated for 72 h with vehicle, 10 nM or 100 nM hGIIA. Cells were stained with Alexa Fluor 647–conjugated anti-PLIN3 (red) or anti-PLIN2 (pseudocolored purple) and DAPI (blue). Violin plots show single-cell PLIN mean intensity representative of three independent experiments, quantified as described in Materials and Methods. *p < 0.05; ***p < 0.001, ****p < 0.0001 (Mann Whitney U test). Scale bars 20 μm. Inset 10 μm.

To determine whether hGIIA-driven changes in LD content were associated with alterations in LD-coat proteins, we next analysed PLIN2 and PLIN3 expression in DU145 WT and DU145^vim−^ cells exposed to 10 or 100 nM hGIIA for 72 h (Figure 4). In DU145 WT cells, PLIN2 protein levels, as determined by Western blot, were largely unchanged at 10 nM hGIIA compared with baseline but were modestly reduced at 100 nM (Figure 4A). In contrast, DU145^vim−^ cells showed a much stronger suppression of PLIN2, with a significant decrease already at 10 nM hGIIA that remained low at 100 nM (Figure 4B).

PLIN3 displayed a distinct, dose- and genotype-dependent pattern. In DU145 WT cells, 10 nM hGIIA induced a robust increase in PLIN3 abundance, while 100 nM hGIIA had no significant effect on PLIN3 relative to baseline (Figure 4C). In DU145^vim−^ cells, 10 nM hGIIA similarly increased PLIN3, but 100 nM hGIIA instead reduced PLIN3 to below basal levels (Figure 4D).

Confocal immunofluorescence using 647-conjugated PLIN3 (red) and PLIN2 (pseudocolored purple) antibodies confirmed these biochemical data: single-cell PLIN3 mean intensity increased at 10 nM hGIIA in both genotypes, with a modest elevation at 100 nM in WT but a marked loss of signal at 100 nM in DU145^vim−^ cells (Figure 4E). PLIN2 staining showed a clear downward trend with a significant reduction at 100 nM in WT cells, whereas DU145^vim−^ PLIN2 signal was reduced at both 10 and 100 nM hGIIA (Figure 4F). Together, these data indicate that hGIIA differentially remodels PLIN2 and PLIN3 in a vimentin-dependent manner. vimentin loss leads to PLIN2 downregulation and converting the high-dose hGIIA response of PLIN3 from sustained induction to suppression.

**FIGURE 5:**
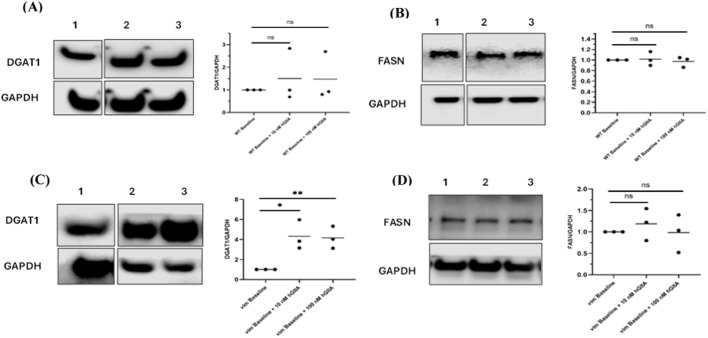
Vimentin loss unmasks hGIIA-induced upregulation of DGAT1 but not FASN. **(A,B)** Representative western blots and quantification of DGAT1 **(A)** and FASN **(B)** normalized to GAPDH in DU145 WT cells treated for 72 h with vehicle (baseline), 10 nM or 100 nM hGIIA. **(C,D)** DGAT1 **(C)** and FASN **(D)** expression in vimentin-knockout DU145^vim−^ cells under the same conditions. horizontal lines indicate the mean. Statistical significance was assessed by unpaired two-tailed Student’s t-test; ns, not significant; *p < 0.05; **p < 0.01. Lane identities are indicated by the numbers shown above each blot and correspond to the treatment groups shown in the graphs (left to right). Non-adjacent lanes were juxtaposed; splicing is indicated by vertical lines; all lanes were from the same membrane and exposure. Uncropped Western blots, with their corresponding molecular markers, are shown in Supplementary Figure S2.

To test whether hGIIA-induced LD remodelling involved changes in lipid-synthesis enzymes, we examined diacylglycerol acyltransferase-1 (DGAT1) and fatty acid synthase (FASN) by Western blot. In DU145 WT cells, DGAT1/GAPDH (Figure 5A) and FASN/GAPDH ratios (Figure 5B) were unchanged at 10 or 100 nM hGIIA, indicating that the biphasic LD response occurs without major alterations in these enzymes. In contrast, vimentin-deficient DU145^vim−^ cells showed a strong induction of DGAT1 in response to hGIIA: both 10 and 100 nM hGIIA significantly increased DGAT1 levels compared with baseline with higher values at 100 nM (Figure 5C). FASN abundance remained unaffected (Figure 5D). These data suggest that, in the absence of vimentin, hGIIA preferentially enhances triglyceride esterification via DGAT1 rather than *de novo* fatty acid synthesis, consistent with the increased LD accumulation observed at high-dose hGIIA in DU145^vim−^ cells.

**FIGURE 6:**
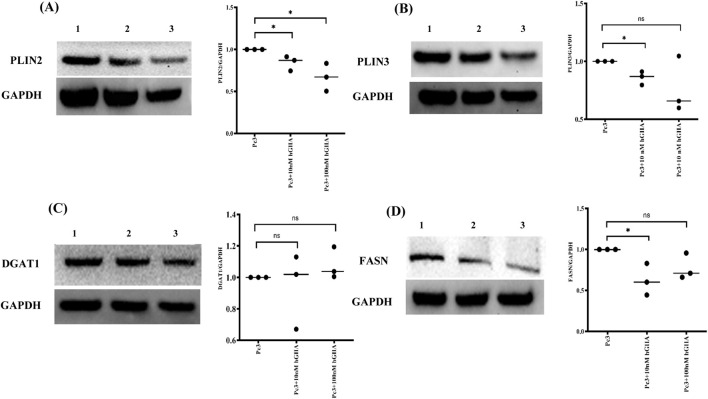
hGIIA reduces PLIN2 and PLIN3 but not DGAT1 or FASN in PC-3 cells. **(A,B)** Representative Western blots and quantification of PLIN2/GAPDH **(A)** and PLIN3/GAPDH **(B)** in PC-3 cells treated for 72 h with vehicle, 10 nM or 100 nM hGIIA. **(C,D)** DGAT1/GAPDH **(C)** and FASN/GAPDH **(D)** under the same conditions. Data points represent independent biological replicates (n = 3); horizontal lines indicate the mean. Statistical significance was assessed by unpaired Student’s t-test; ns, not significant; *p < 0.05; Lane identities are indicated by the numbers shown above each blot and correspond to the treatment groups shown in the graphs (left to right). All lanes were from the same membrane and exposure. Uncropped Western blots, with their corresponding molecular markers, is represented in Supplementary Figure S3.

To test whether the hGIIA–perilipin axis was conserved across PCa lines, we performed the same analysis in PC-3 cells. In PC-3 cells, however, PLIN2 and PLIN3 both showed a clear tendency to decrease at 10 and 100 nM hGIIA (Figures 6A,B). This downward trend remained evident even when accounting for an outlier in the PLIN3 dataset and is compatible with the reduced BODIPY intensity observed in PC-3 in Figure 1. By contrast, DU145 cells displayed a distinct, biphasic response, with PLIN3 induction at low-dose hGIIA and only modest PLIN2 loss at high dose (Figures 4A,C). Consistent with our DU145 data (Figures 5A,B), hGIIA had little effect on the lipogenic enzymes DGAT1 and FASN in either cell line (Figures 6C,D). Together, these data indicate that hGIIA-driven perilipin remodelling occurs in both cell lines, however the effect of exogenous hGIIA is clearly influenced by genetic and metabolic background of the PCa cell.

**FIGURE 7:**
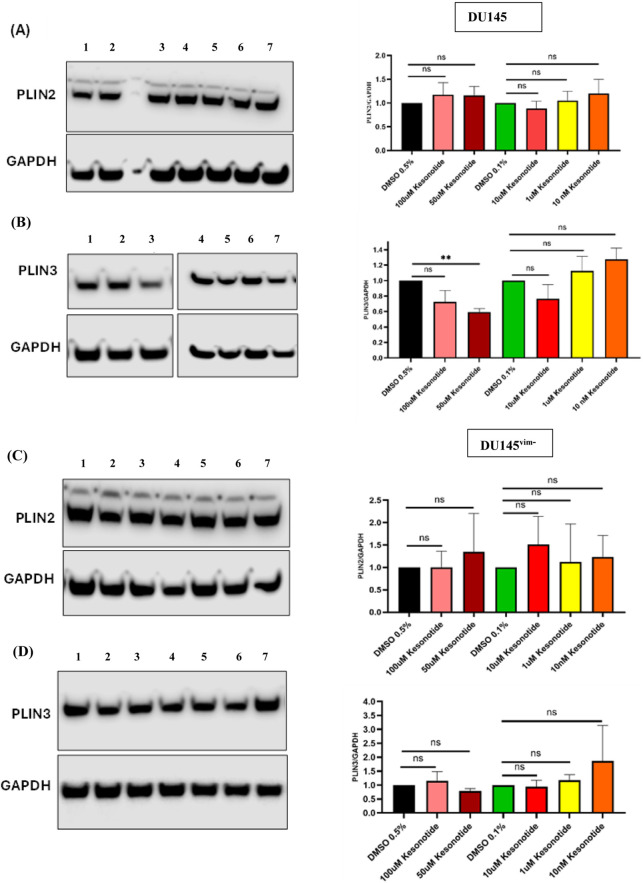
Kesonotide alone has a vimentin-dependent biphasic effect on PLIN3 expression in DU145 cells. **(A,B)** WT DU145 cells: representative western blots and quantification of PLIN2/GAPDH **(A)** and PLIN3/GAPDH **(B)** after 72 h treatment with DMSO 0.5%, 100 µM Kesonotide, 50 µM Kesonotide, DMSO 0.1%, or the indicated lower Kesonotide concentrations. DMSO 0.5% serves as the vehicle control for 100 and 50 µM Kesonotide, and DMSO 0.1% serves as the vehicle control for the remaining concentrations. **(C,D)** Vimentin-knockout DU145^vim−^ cells analysed as in **(A,B)**. Bars show mean ± SEM of three independent experiments. Statistical significance was assessed by unpaired Student’s t-test; ns, not significant; **p < 0.01. Lane identities are indicated by the numbers shown above each blot and correspond to the treatment groups shown in the graphs (left to right). Non-adjacent lanes were juxtaposed; splicing is indicated by vertical lines; all lanes were from the same membrane and exposure. Uncropped Western blots, with their corresponding molecular markers, are shown in Supplementary Figure S4.

Based on our recently published work showing that exogenous hGIIA binds intracellular vimentin and that the non-catalytic inhibitor Kesonotide disrupts this interaction (Mann et al., 2026), we next asked whether Kesonotide itself alters perilipin expression in DU145 cells. In WT DU145 cells, PLIN2/GAPDH expression was not significantly changed at any dose, however, there was a modest trend to increased inhibition with increasing concentration up to 10 μM (Figure 7A) which was lost at higher doses (100 µM 50 µM). Kesonotide showed a clear biphasic response in PLIN3 expression with a trend to inhibition of expression with increasing dose with a significant reduction at 50 µM. In this case, as with PLIN2, the trend was lost at higher dose (100 µM) (Figure 7B). In vimentin-knockout DU145^vim−^ cells, neither PLIN2 nor PLIN3 was altered by Kesonotide at any concentration (Figures 7C,D). Thus, Kesonotide alone has minimal impact on perilipin abundance, apart from a small PLIN3 decrease at 50 µM in WT cells. To assess any off-target effects of Kesonotide on perilipins, we treated DU145 cells (which do not express endogenous hGIIA) with increasing Kesonotide concentrations and measured PLIN2 and PLIN3.

**FIGURE 8:**
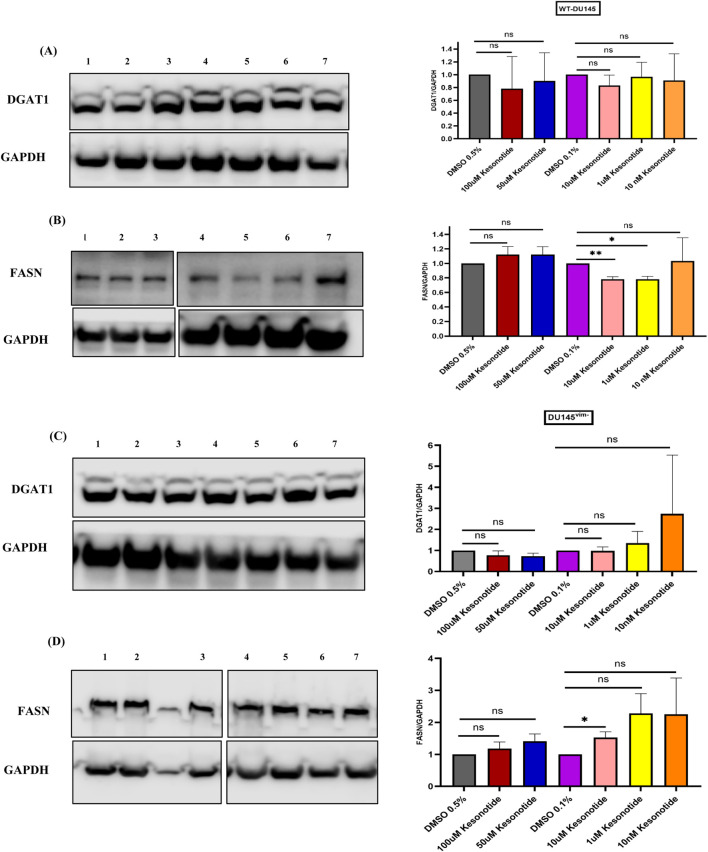
Kesonotide has minimal effects on DGAT1 and FASN expression in DU145 cells. **(A,B)** WT DU145 cells; **(C,D)** vimentin-knockout DU145^vim−^ cells. Representative western blots and quantification of DGAT1/GAPDH **(A,C)** and FASN/GAPDH **(B,D)** after 72 h treatment with DMSO 0.5%, 100 µM Kesonotide, 50 µM Kesonotide, DMSO 0.1%, and the indicated lower Kesonotide concentrations. DMSO 0.5% serves as the vehicle control for 100 and 50 µM Kesonotide, and DMSO 0.1% serves as the vehicle control for the remaining concentrations. Bars show mean ± SEM of three independent experiments. *p < 0.05, **p < 0.01; ns, not significant. Lane identities are indicated by the numbers shown above each blot and correspond to the treatment groups shown in the graphs (left to right). Non-adjacent lanes were juxtaposed; splicing is indicated by vertical lines; all lanes were from the same membrane and exposure. Uncropped Western blots, with their corresponding molecular markers, are shown in Supplementary Figure S5.

We next asked whether Kesonotide alone modulates enzymes involved in triglyceride synthesis. In WT DU145 cells, DGAT1/GAPDH levels were unchanged across all Kesonotide concentrations compared with their DMSO controls (Figure 8A). FASN/GAPDH showed only modest changes, with a small but significant reduction at 10 µM Kesonotide relative to 0.1% DMSO, whereas other doses were not significantly different from vehicle (Figure 8B). In vimentin-knockout DU145^vim−^ cells, Kesonotide again had little impact: DGAT1/GAPDH remained stable at all concentrations (Figure 8C), and FASN/GAPDH showed only a minor increase at 1 µM versus the 0.1% DMSO control without a consistent dose–response trend (Figure 8D). Together with the perilipin data, these results indicate that Kesonotide on its own does not robustly alter core lipogenic enzymes in DU145 cells, supporting that the strong DGAT1 induction observed in vimentin-deficient cells is specific to exogenous hGIIA rather than an off-target effect of the inhibitor.

**FIGURE 9:**
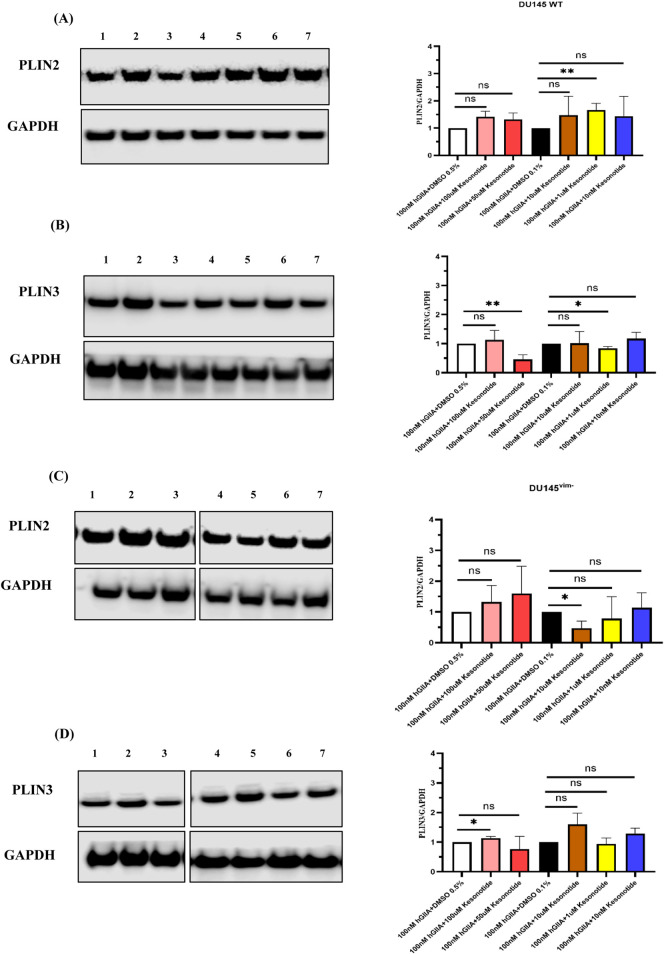
Kesonotide modulates PLIN2 and PLIN3 levels in the presence of high-dose hGIIA. **(A,B)** DU145 WT cells; **(C,D)** vimentin-knockout DU145^vim−^ cells. Representative western blots and quantification of PLIN2/GAPDH **(A,C)** and PLIN3/GAPDH **(B,D)** 100 nM hGIIA in combination with DMSO 0.5% serves as the vehicle control for 100 and 50 µM Kesonotide, and with DMSO 0.1% serves as the vehicle control for the remaining concentrations. Bars show mean ± SEM of three independent experiments. Statistical significance was assessed by unpaired Student’s t-test; ns, not significant, *p < 0.05, **p < 0.01. Lane identities are indicated by the numbers shown above each blot and correspond to the treatment groups shown in the graphs (left to right). Non-adjacent lanes were juxtaposed; splicing is indicated by vertical lines; all lanes were from the same membrane and exposure. Uncropped Western blots, with their corresponding molecular markers, are shown in Supplementary Figure S6.

Kesonotide co-treatment partially restored PLIN abundance: PLIN2 and PLIN3 levels increased toward control. Combination treatments were performed at a single hGIIA dose (100 nM) to approximate near-saturating vimentin occupancy and to maximize the dynamic range for detecting Kesonotide-mediated modulation. In DU145 WT cells, the modest PLIN2 reduction induced by 100 nM hGIIA alone (Figure 4A) was no longer evident when Kesonotide was co-applied: PLIN2/GAPDH values in all Kesonotide combinations were not significantly different from their respective DMSO controls (Figure 9A). PLIN3 showed a similar pattern - despite some small pairwise differences between Kesonotide doses, overall levels clustered around the DMSO controls and no clear suppressive effect of 100 nM hGIIA remained (Figure 9B). In DU145^vim−^ cells, 100 nM hGIIA alone strongly reduced both PLIN2 and PLIN3 (Figures 4B,D) values and critically, none of the Kesonotide–hGIIA combinations differed significantly from their matched DMSO controls (Figures 9C,D). Thus, although Kesonotide alone has little impact on PLINs, in the presence of 100 nM hGIIA it effectively neutralizes the PLIN2/PLIN3-remodelling seen with hGIIA alone, consistent with Kesonotide disrupting the hGIIA–vimentin axis that drives these changes.

**FIGURE 10:**
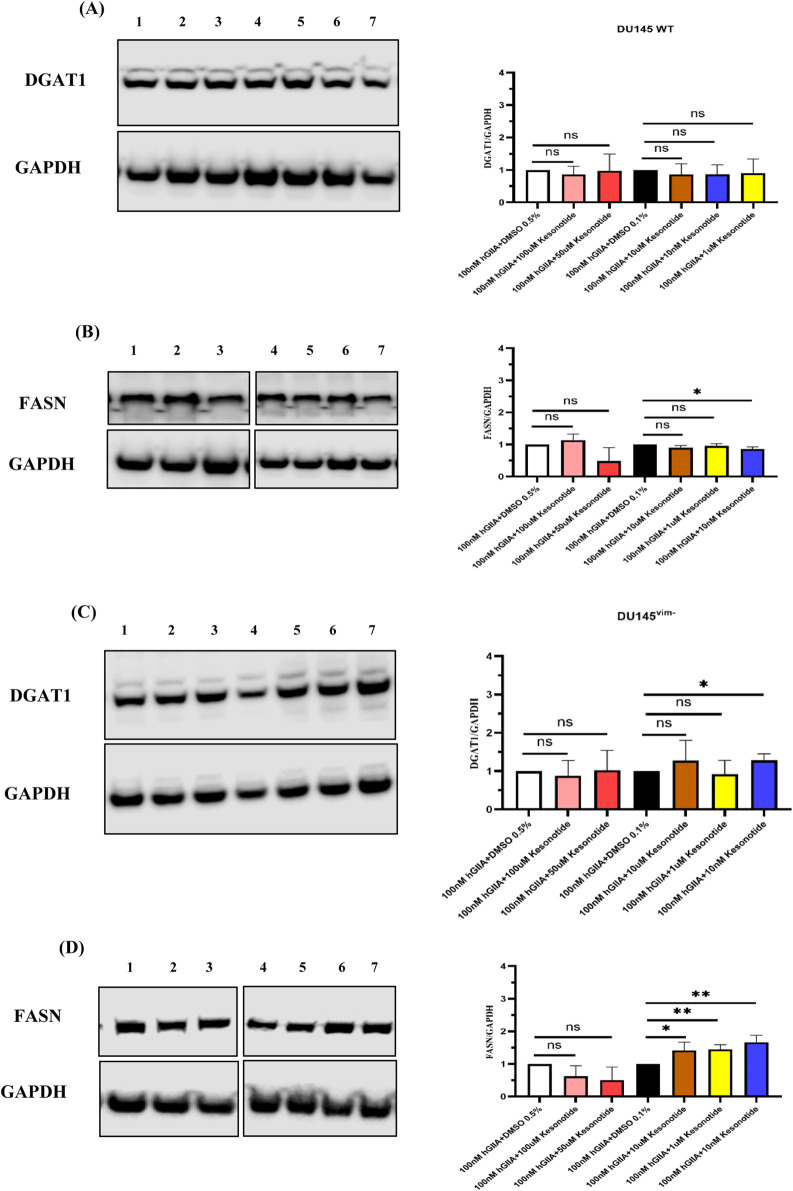
Kesonotide modulates DGAT1 and FASN expression in DU145 cells exposed to high-dose hGIIA. **(A,B)** DU145 WT cells; **(C,D)** vimentin-knockout DU145^vim−^ cells. Representative western blots and quantification of DGAT1/GAPDH **(A,C)** and FASN/GAPDH **(B,D)** after 72 h treatment with 100 nM hGIIA plus DMSO 0.5% serves as the vehicle control for 100 and 50 µM Kesonotide and with DMSO 0.1% serves as the vehicle control for the remaining concentrations. Bars show mean ± SEM of three independent experiments. Statistical significance was assessed by unpaired Student’s t-test; ns, not significant; *p < 0.05; **p < 0.01. Lane identities are indicated by the numbers shown above each blot and correspond to the treatment groups shown in the graphs (left to right). Non-adjacent lanes were juxtaposed; splicing is indicated by vertical lines; all lanes were from the same membrane and exposure. Uncropped Western blots, with their corresponding molecular markers, are shown in Supplementary Figure S7.

Given that 100 nM hGIIA alone strongly induced DGAT1 in DU145^vim−^ cells but not in WT cells (Figure 5), and that Kesonotide by itself had only minor effects on DGAT1 and FASN (Figure 8), we next examined how Kesonotide modifies the lipogenic response to high-dose hGIIA. In DU145 WT cells, DGAT1/GAPDH remained unchanged across all 100 nM hGIIA + Kesonotide combinations, consistent with the lack of effect seen with either hGIIA or Kesonotide alone (Figure 10A). FASN/GAPDH showed only small, non-monotonic changes, with a modest reduction at one intermediate Kesonotide concentration but no consistent dose–response pattern (Figure 10B).

In vimentin-deficient DU145^vim−^ cells, co-treatment with Kesonotide partially reshaped the hGIIA-driven program. The strong DGAT1 upregulation observed with 100 nM hGIIA alone (Figure 5C) was attenuated in the presence of Kesonotide, with DGAT1 levels reduced toward those seen under Kesonotide -only conditions and only a single Kesonotide dose differing significantly from the DMSO control (Figure 10C). By contrast, FASN, which was not altered by hGIIA alone (Figure 5D) and showed only minor changes with Kesonotide alone (Figure 8D), now tended to increase in several Kesonotide–hGIIA combinations, with significant elevations at intermediate Kesonotide concentrations (Figure 10D). Together, these data suggest that disruption of the hGIIA–vimentin axis by Kesonotide dampens DGAT1 induction while permitting a modest compensatory rise in FASN in DU145^vim−^ cells exposed to high-dose hGIIA.

## 4 Discussion

This study identifies a previously unrecognized role for exogenous hGIIA in controlling LD biogenesis and remodelling in PCa cells (Figure 1) and establishes vimentin as a key determinant of LD formation (Figure 2) and the exogenous hGIIA response (Figure 3). We show that the hGIIA-vimentin axis rewires LD dynamics, perilipin expression, and triglyceride esterification enzyme expression in a dose-, genotype- and cell line–dependent manner (Figures 3–10). These data extend hGIIA biology beyond its classical secreted phospholipase enzymatic function and position vimentin intermediate filaments as a regulator that may shape how PCa cells deploy LDs under lipid and inflammatory stress.

The loss of robust OA-dependent increase in LD-associated BODIPY signal on vimentin deletion (Figure 2) indicates that vimentin is not only a structural component but actively supports the assembly and/or stabilization of LDs in response to excess fatty acid supply. Vimentin intermediate filaments associate with and organize intracellular organelles, including LDs, in multiple cell types (Nekrasova et al., 2011; Heid et al., 2014; Klemm and Carvalho, 2024). The failure of vim^−^ cells to increase LD content in response to OA suggests that vimentin is required for efficient coupling between exogenous fatty acid supply and LD biogenesis, potentially by supporting the structural organization of LDs and associated metabolic machinery. However, the precise subcellular mechanisms by which vimentin regulates OA-driven LD formation remain to be determined.

The observations that low dose hGIIA addition to WT DU145 cells resulted in increased LD intensity (Figure 3A), increased PLIN3 expression (Figure 4B) with no effect on lipid synthesis enzyme expression (Figures 5A,B) imply that low dose hGIIA increases nascent early LD stability, resulting in increased lipid droplet intensity through perilipin remodelling rather than through upregulating lipid biosynthetic pathway enzymes. PLIN3 is typically associated with nascent LDs and dynamic remodelling, whereas PLIN2 stabilizes more mature droplets (Choi et al., 2023; Xu et al., 2019; Olzmann and Carvalho, 2019). The combination of preserved PLIN2 and induced PLIN3 is therefore consistent with controlled expansion of the LD compartment. Vimentin loss abrogates the hGIIA-mediated increase in LD intensity (Figure 3D), likely through destabilization of mature LDs due to loss of the protective effect of PLIN2 (Figure 4C) despite a compensatory increase in DGAT1 expression (Figure 5C). PLIN2 normally physically shields neutral lipids in LDs and its loss results in reduced LD half-life and faster clearance (Tsai et al., 2017).

At high hGIIA, we hypothesise that a similar mechanism as described above for vimentin loss at low dose hGIIA can explain how high dose hGIIA reduces LD intensity due to decreasing PLIN2 expression (Figure 4A) with no change in PLIN3 (Figure 4B). These effects could result in destabilization of mature LDs even in the presence of the protective effect of vimentin, though without any compensatory increase in DGAT1 expression (Figure 5A). However, the loss of the stabilising effect of vimentin under the conditions of high dose hGIIA, likely pushes the cell over a stress threshold that results in sublethal but chronic membrane instability resulting in lipotoxicity signals. These signals could cause the cell to flip from the homeostatic buffering of cellular lipid metabolism through regulating storage in LDs (low dose hGIIA in WT DU145 and DU145^vim−^ cells and low dose hGIIA in DU145^vim−^) to rapid LD synthesis by upregulating lipid flux through the DGAT pathway as a containment strategy (high dose hGIIA, DU145^vim−^). This striking genotype-specific “flip” suggests that vimentin dictates how hGIIA signals are interpreted at the level of lipid storage. Though not conclusive, with respect to mechanism, the current study demonstrates that lipid droplet formation is a dynamic vimentin-dependent process that is responsive to stress induced by factors such as hGIIA present in the tumour microenvironment and lays the groundwork for further research to robustly test these ideas.

Changes in LD-associated lipid biosynthetic enzymes further refine this model. In DU145 WT cells, DGAT1 and FASN levels were unchanged across hGIIA doses (Figure 5). In vimentin-deficient DU145^vim−^ cells, however, hGIIA induced a strong, dose-dependent increase in DGAT1, while FASN remained stable. We hypothesise that, in the absence of vimentin, hGIIA preferentially enhances triglyceride esterification capacity rather than *de novo* fatty acid synthesis, aligning with the pronounced LD accumulation observed at high-dose hGIIA (Figure 3C). Further, although the phospholipase activity of intracellular hGIIA does not function due to insufficient calcium inside cells (Mann et al., 2026) indirect hGIIA-mediated phospholipid hydrolysis via activation of cPLA_2_-α (Mann et al., 2026; Guijas et al., 2014; Jarc and Petan, 2020) may increase availability of fatty acids or lysophospholipid-derived intermediates, which could be channelled into DGAT1-dependent triglyceride synthesis. DGAT1-dependent LD biogenesis supports stress tolerance and survival in several cancers (Cheng et al., 2020; Wilcock et al., 2022; He P et al., 2021; Petan et al., 2018) and given that hGIIA is an inflammatory, tumour-microenvironment enzyme upregulated in multiple malignancies (Scott et al., 2021), our finding that hGIIA robustly induces DGAT1 in vimentin-deficient DU145 cells suggests a previously unrecognized hGIIA - DGAT1 axis that could promote lipid-based adaptation in this context. It should be noted however, that our Western blot data, while suggestive of enhanced lipid flux through DGAT1, do not directly measure triglyceride esterification capacity and further work is necessary to definitively show this.

The BODIPY staining patterns in DU145 versus PC-3 (Figure 1) are consistent with cell line-specific differences in neutral-lipid organization (Sorvina et al., 2018) which can result in a shift from a more diffuse/reticular cytoplasmic pool to a more punctate, compartmentalized LD pattern. In PC-3 cells, both PLIN2 and PLIN3 tended to decrease at 10 and 100 nM hGIIA, mirroring the reduced BODIPY intensity observed in these cells (Figure 1). This contrasts with the biphasic PLIN3 induction and relatively modest PLIN2 loss in DU145 WT cells. Importantly, DGAT1 and FASN remained largely unchanged in both lines under hGIIA treatment, indicating that the pronounced DGAT1 upregulation we observed is specific to vimentin-deficient cells rather than being a general property of PCa lines.

The field of cancer biomarkers is rapidly evolving at present, driven largely by correlative analysis of patient outcomes with features derived from bioinformatic analysis of single cell and bulk tissue ‘omics’ data. Functional studies such as those described here highlight that subtle quantitative changes in the abundance of putative biomarkers such as hGIIA or vimentin, may have dramatic and context-dependent functional consequences for cell metabolism and consequently treatment outcomes, highlighting the need to carefully consider such changes when stratifying patients based on them.

The Kesonotide experiments provide important mechanistic reassurance and further delineate the specificity of the hGIIA–vimentin axis in LD modulation. Kesonotide alone produced only minor, non-monotonic changes in PLIN2 and PLIN3 in DU145 WT cells, with a small PLIN3 decrease at 50 µM and no consistent effects at other doses (Figures 7A,B). In DU145^vim−^ cells, perilipin levels were unaffected (Figures 7C,D). Similarly, DGAT1 levels remained stable in both genotypes, and FASN showed only a small reduction at 10 µM Kesonotide in WT cells and a minor, non-dose-dependent increase at 1 µM in vimentin-knockout cells (Figure 8). Given that DU145 cells do not express endogenous hGIIA, these data indicate that Kesonotide has minimal off-target activity on perilipins or core lipogenic enzymes in the absence of hGIIA. Consequently, the strong DGAT1 induction (Figure 5) and LD remodelling (Figure 3.) observed in vimentin-deficient cells can be attributed to exogenous hGIIA itself, rather than to the inhibitor. More broadly, this suggests that pharmacological disruption of the hGIIA–vimentin interaction is unlikely to grossly perturb basal LD biology when hGIIA levels are low, which is encouraging from a therapeutic safety perspective.

The combination experiments with Kesonotide further refine this model by functionally testing how disruption of the hGIIA–vimentin interaction feeds back on LD remodelling and lipogenic enzyme expression. In WT DU145 cells, where 100 nM hGIIA alone produced only a modest PLIN2 reduction and little net effect on PLIN3, co-treatment essentially normalized perilipin levels. PLIN2 and PLIN3 expression across all kesonotide-hGIIA combinations were indistinguishable from their respective DMSO controls (Figure 9). A similar “normalization” was seen in the vimentin-knockout DU145^vim−^ line, in which 100 nM hGIIA alone strongly suppressed both PLIN2 and PLIN3. Here, addition of kesonotide partially restored PLIN2/PLIN3 abundance towards basal values, and none of the combination treatments differed significantly from the corresponding vehicle controls. These data indicate that kesonotide does not simply add a small effect on top of hGIIA signalling but instead effectively neutralizes the perilipin remodelling program driven by high-dose hGIIA in both genotypes. In the context of our previous work showing that kesonotide disrupts hGIIA–vimentin binding (Mann et al., 2026), this strongly supports the idea that hGIIA’s ability to remodel PLIN2 and PLIN3 is contingent on its non-catalytic interactions.

The effect of Kesonotide on DGAT1 and FASN extend this conclusion to the level of lipid-synthesis enzymes, particularly in vimentin-deficient cells. DGAT1 levels in combination-treated cells regressed towards those observed under Kesonotide - only conditions. Only one Kesonotide dose remained significantly elevated relative to DMSO (Figure 10). Notably, under these same conditions FASN, which was stable under hGIIA alone and only modestly affected by Kesonotide alone, showed a tendency to increase, with significant upregulation at several intermediate Kesonotide concentrations. This pattern suggests that blocking hGIIA’s non-catalytic interactions does not simply shut down all lipogenic responses but selectively dampens DGAT1-driven triglyceride esterification while unmasking a modest, compensatory increase in FASN expression. In vimentin-deficient DU145^vim−^ cells, Kesonotide therefore appears to re-balance the hGIIA-driven lipogenic program away from a DGAT1-dominant, LD-expansion phenotype towards a more evenly distributed lipogenesis profile. While our enzyme expression data suggests this, it will be necessary to confirm that these changes in expression translates to the expected changes in lipid metabolism directly.

Several limitations of this work point to future directions. Our experiments were performed *in vitro* and in two PCa lines. Extending these analyses to additional models with defined vimentin and lipid metabolic status will be important to determine the generality of the hGIIA–vimentin–LD axis. We did not directly measure lipid flux, triglyceride synthesis rates, or lipophagy, so the precise contribution of DGAT1 activity, LD turnover, and autophagic pathways remains to be established. It will also be informative to test whether pharmacological inhibition or genetic knockdown of DGAT1 can blunt high-dose hGIIA-induced LD accumulation in vimentin-deficient cells, and whether hGIIA and/or Kesonotide treatment modify LD dynamics and cell survival under conditions of cell stress. Our cyclic peptide inhibitor of the hGIIA/vimentin interaction limits the effect of hGIIA on LD protein expression in these cellular models, however the generality of Kesonotide effects in other cell systems where lipid metabolic rewiring occurs is currently uncertain.

**FIGURE 11:**
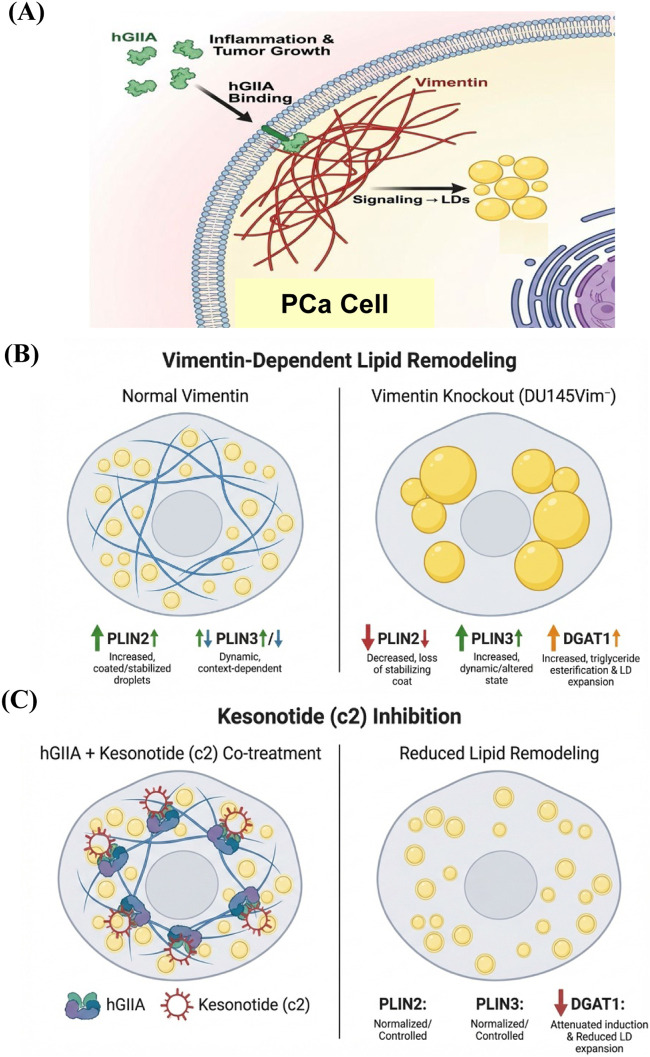
hGIIA–vimentin coupling drives lipid droplet (LD) remodelling in prostate cancer cells and is attenuated by Kesonotide (c2). **(A)** Mechanism. Extracellular hGIIA is internalized and binds to vimentin in the PCa cell, which triggers intracellular signalling that converges on LD pathways, with intracellular vimentin depicted as a key scaffold shaping the downstream response. **(B)** Vimentin-dependent lipid remodelling. In vimentin-competent cells, on hGIIA addition, LD markers appear more organised than in Vim^−^ cells with induction of PLIN2 and regulation of PLIN3 expression in a dynamic and dose-sensitive manner. In DU145Vim− cells, LDs enlarge/coalesce with reduced PLIN2, increased PLIN3, and increased DGAT1. **(C)** Kesonotide inhibition. Co-treatment with hGIIA + Kesonotide (c2) dampens the hGIIA–vimentin axis, resulting in reduced LD remodelling, with PLIN2/PLIN3 shown as controlled/normalised and DGAT1 reduced. This schematic summarizes the putative mechanism proposed in this study. It was created specifically for this article using FigureLabs (figurelab.ai) from an author-written prompt and subsequently edited by the authors. To our knowledge, it is original to this manuscript and is not adapted from any specific previously published Figure.

In summary, our study establishes that the secreted innate immune mediator hGIIA, when added exogenously, modulates LD metabolism in cellular models of advanced PCa, establishes a role for vimentin in regulating LD formation and highlights the hGIIA-vimentin axis as a mechanism by which this immune modulator may influence lipid metabolic rewiring in PCa cells. Our working model (Figure 11) is that extracellular hGIIA engages PCa cells and through coupling to intracellular vimentin, rewires lipid-droplet biogenesis and LD-coat composition. By disrupting the hGIIA–vimentin interaction, Kesonotide dampens downstream LD remodelling (reflected by perilipin normalization and reduced DGAT1-driven triglyceride storage), providing a mechanistic link between inflammatory signalling and lipid metabolic reprogramming in aggressive prostate cancer.

## Data Availability

The original contributions presented in the study are included in the article/Supplementary Material, further inquiries can be directed to the corresponding author.
