# Supplementary material for: The innate immune mediator group IIA secreted phospholipase A2 modulates lipid droplet formation in prostate cancer cells
Source: Front Cell Dev Biol. 2026 May 18;14:1774027. doi: 10.3389/fcell.2026.1774027 (PMC13222947; doi:10.3389/fcell.2026.1774027)
Supplement: Supplementary file 1 [file DataSheet1.pdf]

Supplementary Data.

Figure S1.

Sample ID.

1	2	3
Control	hGIIA (10 nM)	hGIIA (100 nM)

Fig.4A DU145 WT Sample 1 – 3.

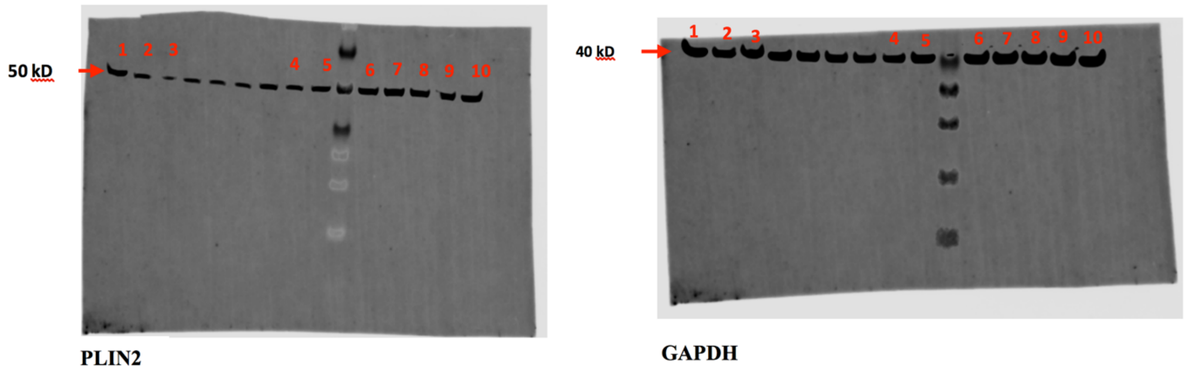

Fig. 4B DU145^{vim-} sample 1 -3.

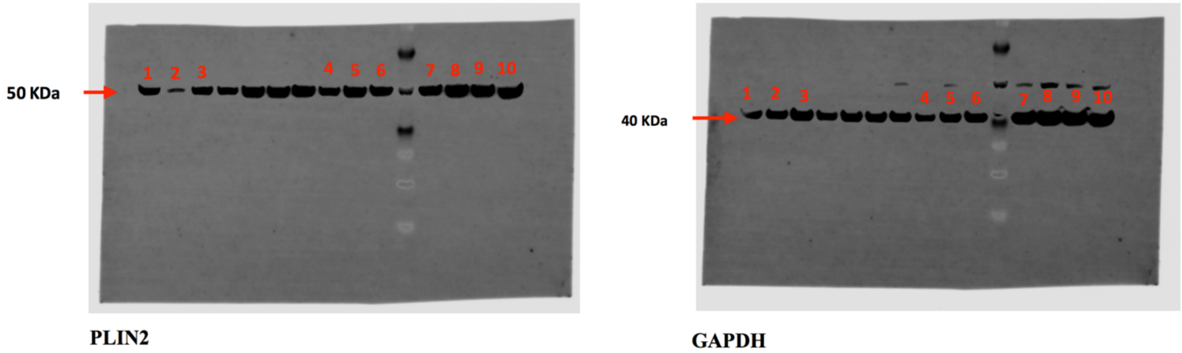

Fig. 4C DU145 WT

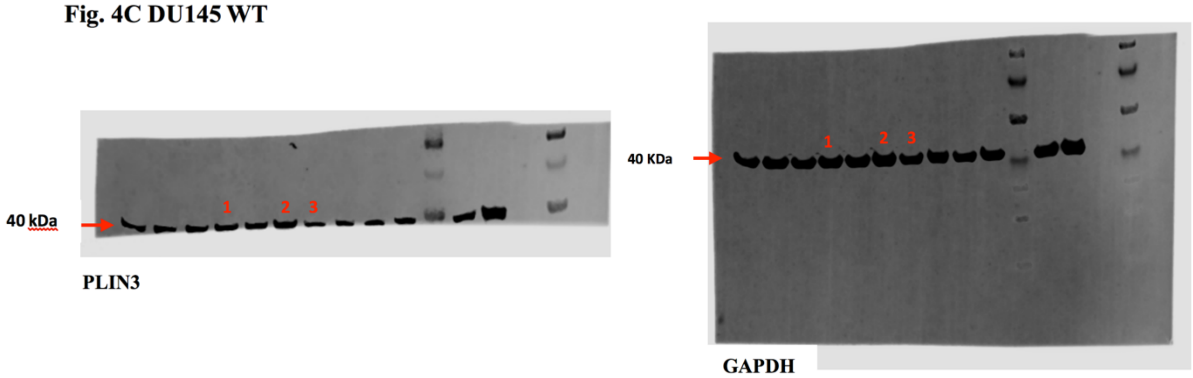

Fig. 4D DU145^{vim-} sample 1 - 3

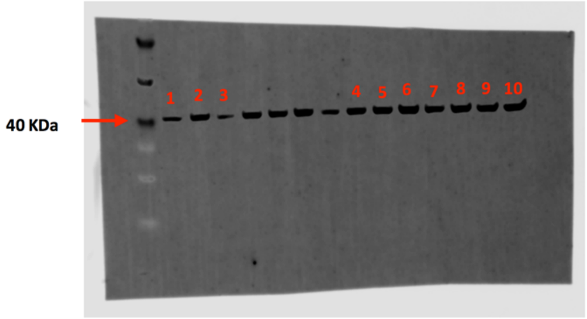

PLIN3

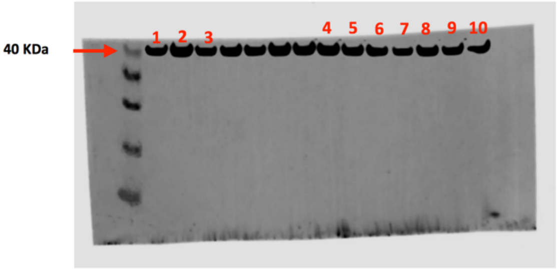

GAPDH

Figure S2

Sample ID

1	2	3
Control	hGIIA (10 nM)	hGIIA (100 nM)

Fig. 5A DU145WT DGAT1 sample 1-3.

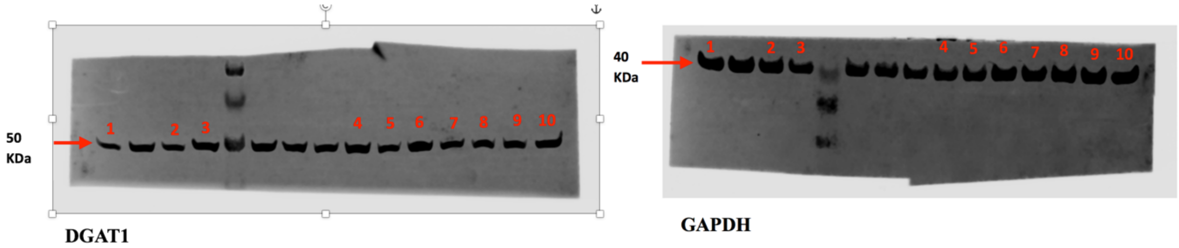

Fig. 5B DU145WT FASN samples 1-3.

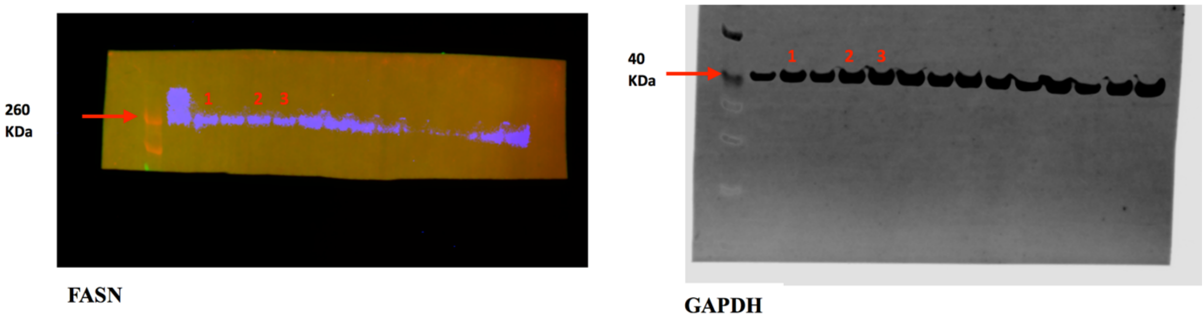

Fig 5C DU145^{vim-} DGAT1 samples 1-3

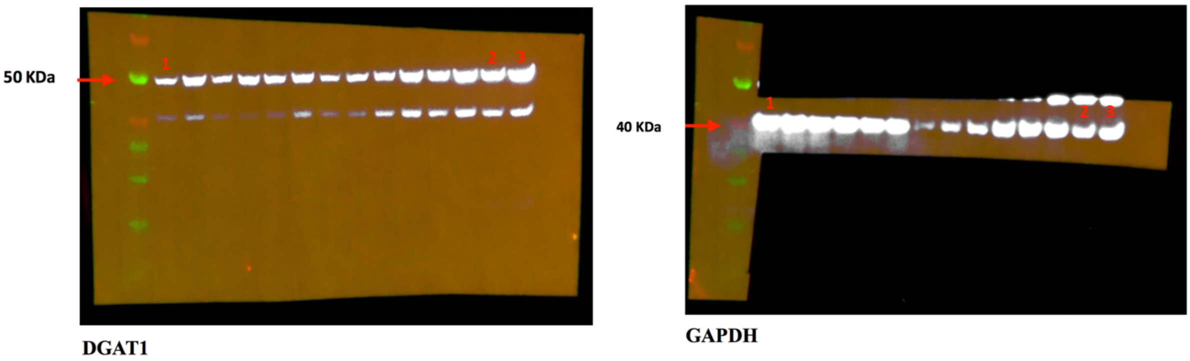

Fig 5D DU145^{vim-} FASN samples 1-3

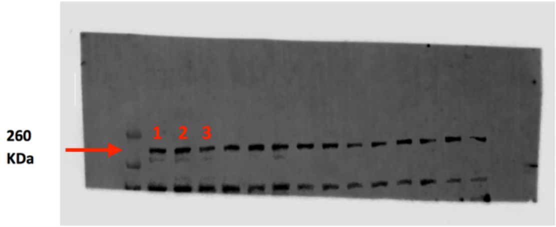

FASN

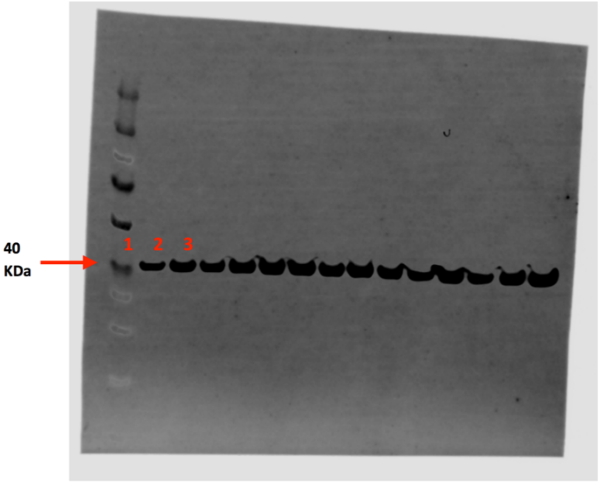

GAPDH

Figure S3.

Sample ID

1	2	3
Control	hGIIA (10 nM)	hGIIA (100 nM)

Fig. 6A PC-3 PLIN2 samples 1-3.

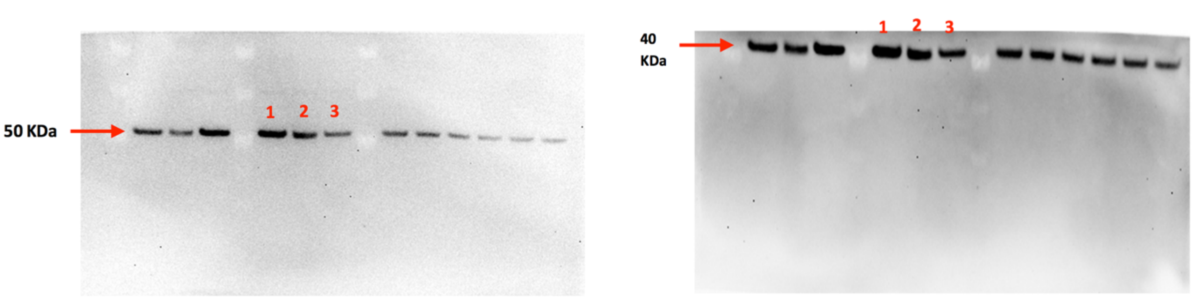

Fig. 6B PC-3 PLIN3 samples 1-3.

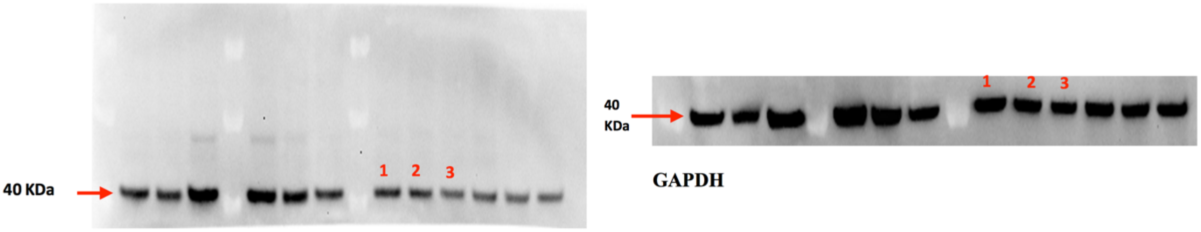

Fig 6C PC-3 DGAT1 samples 1-3.

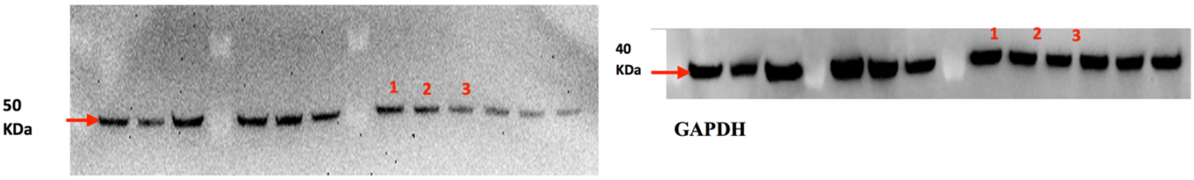

Fig 6D PC-3 FASN samples 1-3.

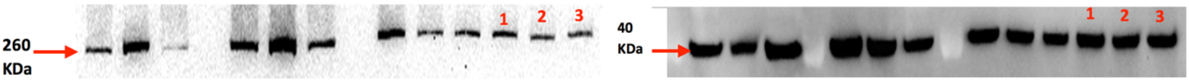

Figure S4.

Sample ID

4	5	6	7	8	9	10
DMSO (0.5%)	Kesonotide (100 µM)	Kesonotide (50 µM)	DMSO (0.1%)	Kesonotide (10 µM)	Kesonotide (1 µM)	Kesonotide (10 nM)

Fig 7A. DU145WT PLIN2 samples 4-10

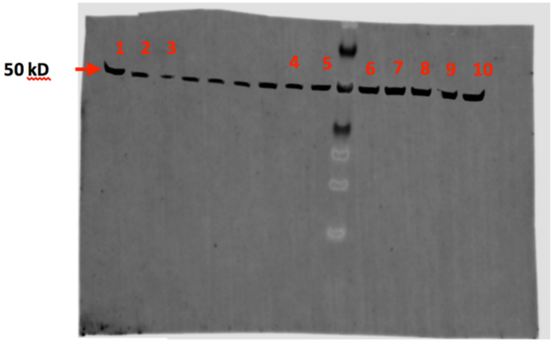

PLIN2

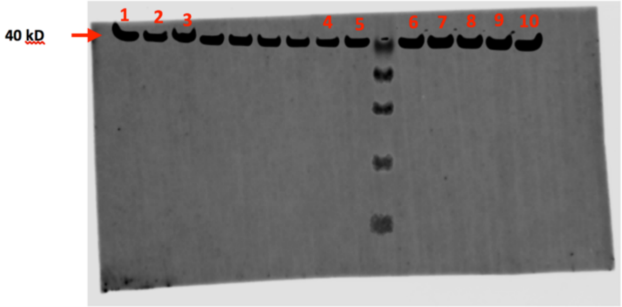

GAPDH

Fig. 7B. DU145WT PLIN3 samples 4-10

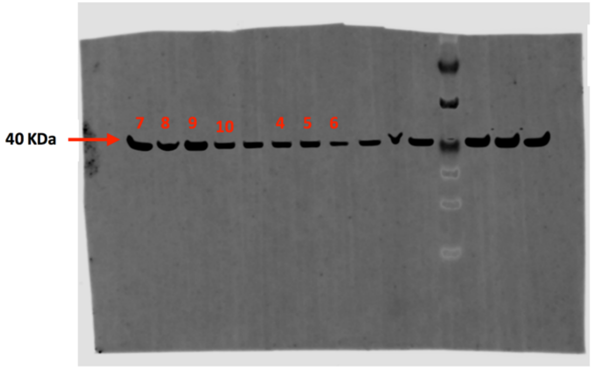

PLIN3

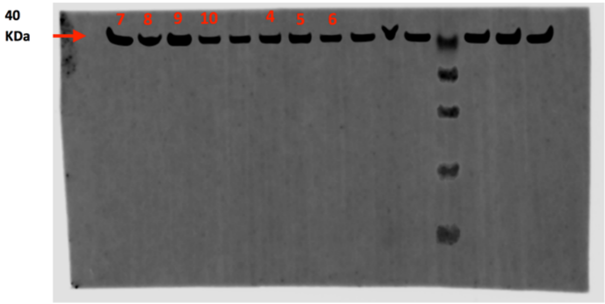

GAPDH

Fig. 7C. DU145^{vim}- PLIN2 samples 4-10

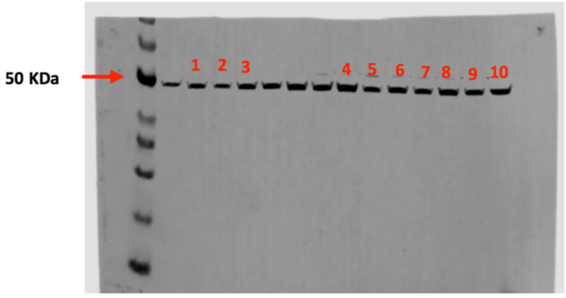

PLIN2

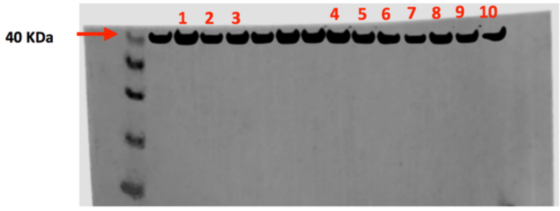

GAPDH

Fig. 7D. DU145^{vim-} PLIN3 samples 4-10

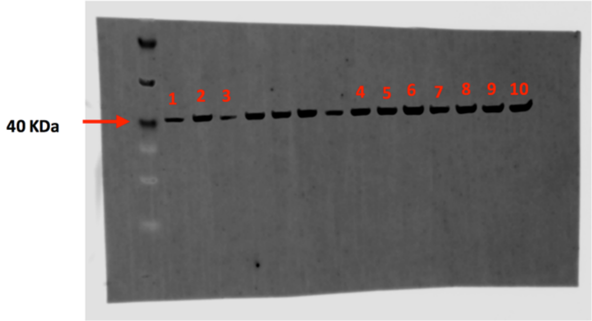

PLIN3

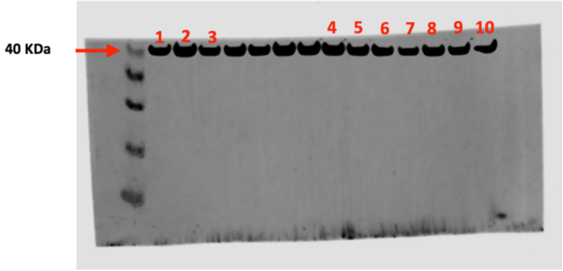

GAPDH

Figure S5.

Sample ID

4	5	6	7	8	9	10
DMSO (0.5%)	Kesonotide (100 µM)	Kesonotide (50 µM)	DMSO (0.1%)	Kesonotide (10 µM)	Kesonotide (1 µM)	Kesonotide (10 nM)

Fig. 8A. DU145WT DGAT1 samples 4-10

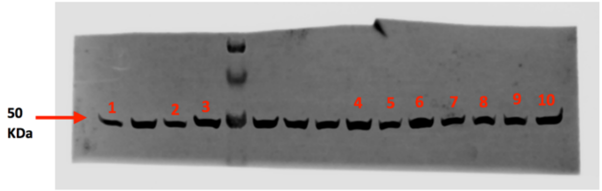

DGAT1

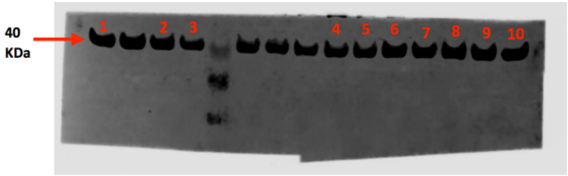

GAPDH

Fig. 8B. DU145WT FASN samples 4-10.

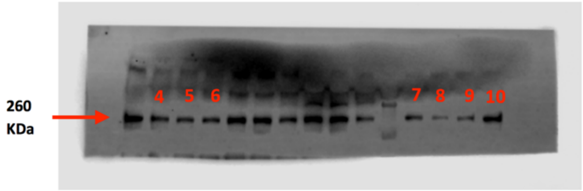

FASN

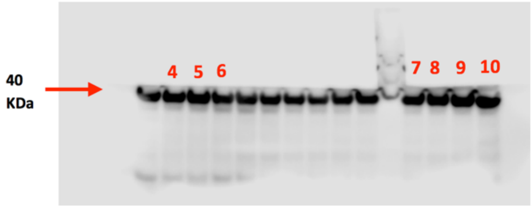

GAPDH

Fig. 8C. DU145^{vim-} DGAT1 samples 4-10

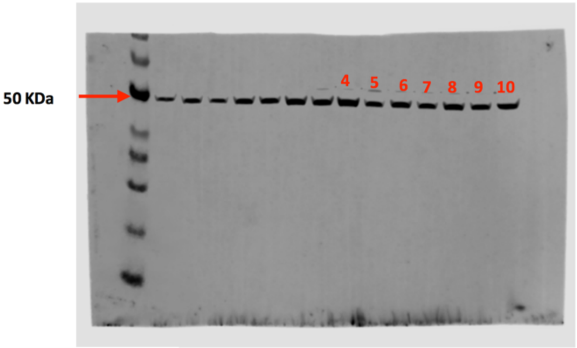

DGAT1

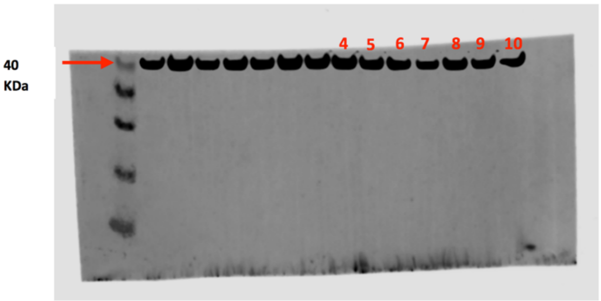

GAPDH

Fig 8D. Du145^{vim-} FASN samples 4-10

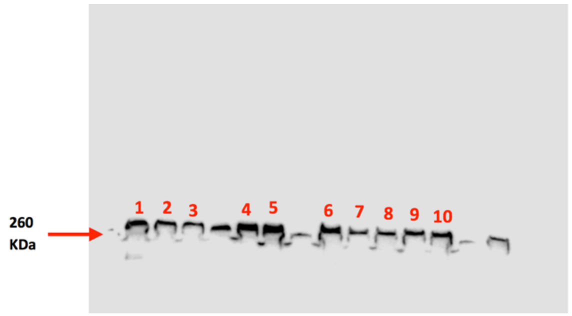

FASN

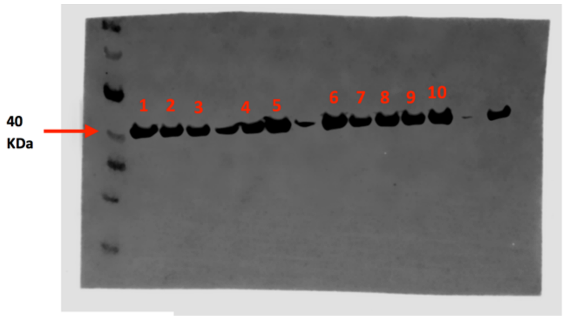

GAPDH

Figure S6.

Sample ID.

1	2	3	4	5	6	7
hGIIA<br>(100nM)<br>DMSO<br>(0.5 %)	hGIIA<br>(100 nM)<br>kesonotide<br>(100 µM)	hGIIA<br>(100 nM)<br>kesonotide<br>(50 µM)	hGIIA<br>(100nM)<br>DMSO<br>(0.5 %)	hGIIA<br>(100 nM)<br>kesonotide<br>(10 µM)	hGIIA<br>(100 nM)<br>kesonotide<br>(1 µM)	hGIIA<br>(100 nM)<br>kesonotide<br>(10 nM)

Fig. 9A DU145WT PLIN2 samples 1-7

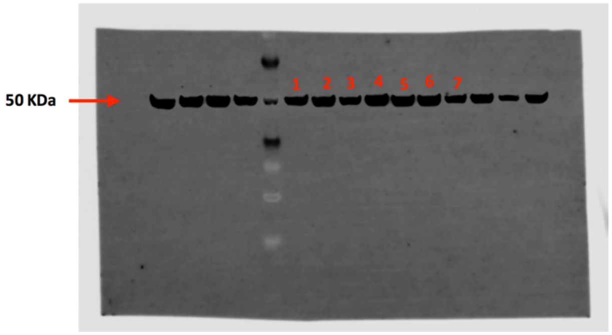

PLIN2

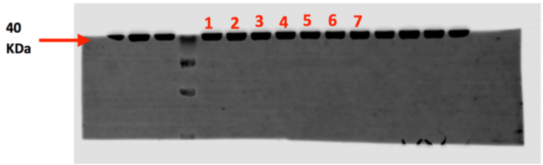

GAPDH

Fig 9B DU145WT PLIN3 samples 1-7

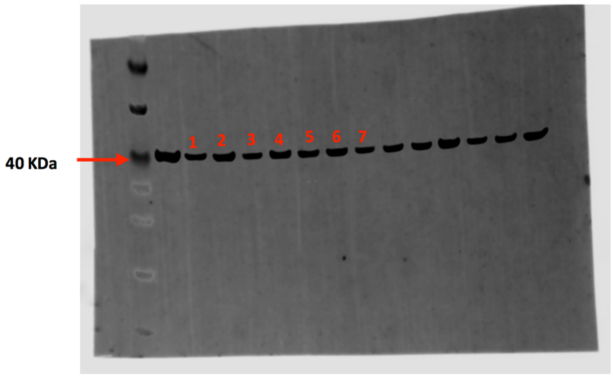

PLIN3

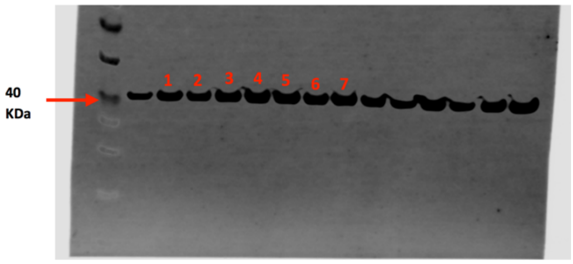

GAPDH

Fig. 9C DU145^{vim-} PLIN2 samples 1-7

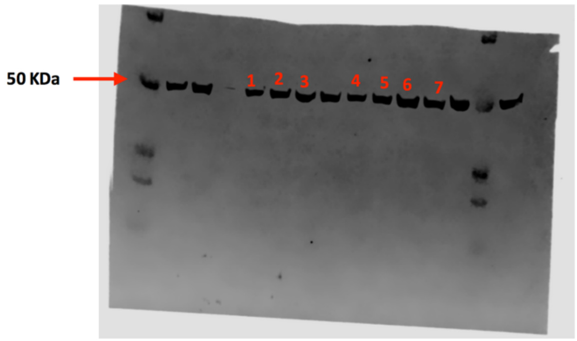

PLIN2

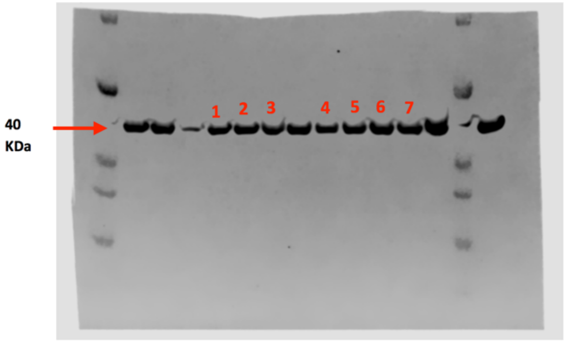

GAPDH

Fig 9D DU145^{vim-} PLIN3 samples 1-7

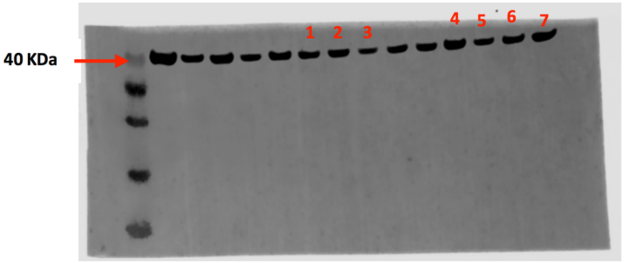

PLIN3

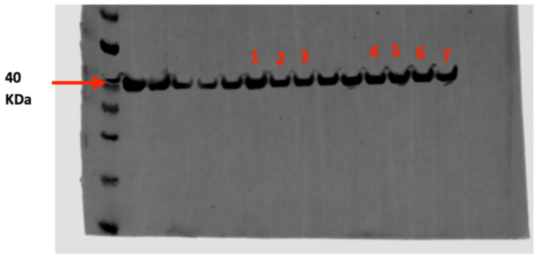

GAPDH

Figure S7

Sample ID.

1	2	3	4	5	6	7
**hGIIA (100nM) DMSO (0.5 %)**	**hGIIA (100 nM) kesonotide (100 μM)**	**hGIIA (100 nM) kesonotide (50 μM)**	**hGIIA (100nM) DMSO (0.5 %)**	**hGIIA (100 nM) kesonotide (10 μM)**	**hGIIA (100 nM) kesonotide (1 μM)**	**hGIIA (100 nM) kesonotide (10 nM)**

Fig. 10A. DU145WT DGAT1 samples 1-7

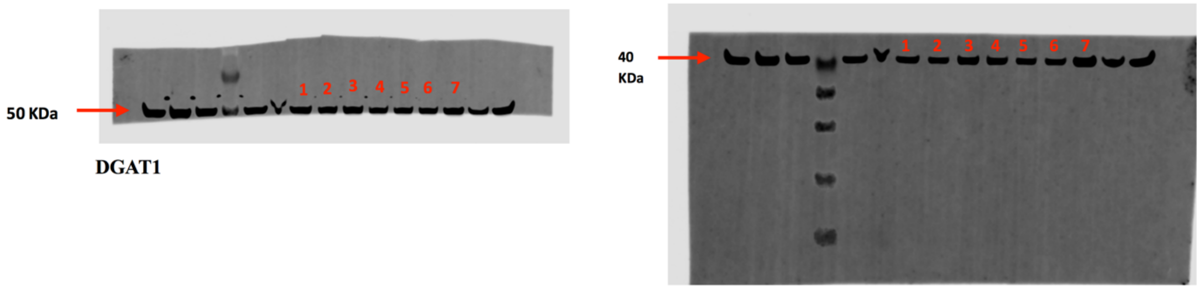

DGAT1

GAPDH

Fig. 10B DU145WT FASN samples 1-7

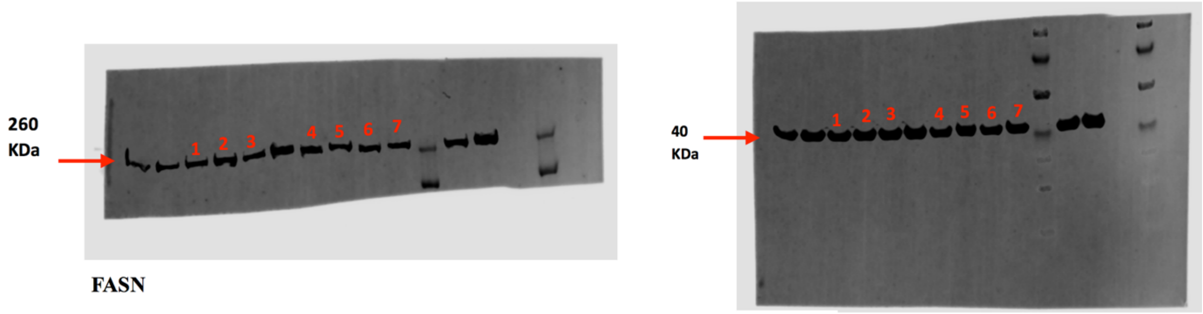

FASN

GAPDH

Fig. 10C DU145^{vim-} DGAT1 samples 1-7

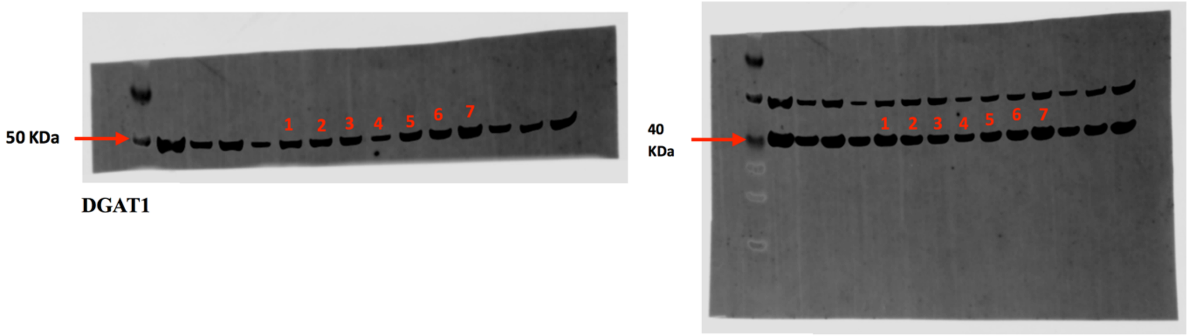

DGAT1

GAPDH

Fig. 10D DU145^{vim-} FASN samples 1-7.

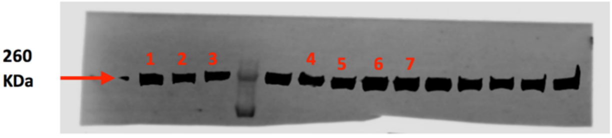

FASN

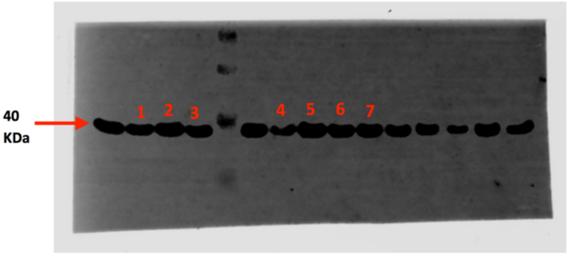

GAPDH
